# In human astrocytes neurotropic flaviviruses increase autophagy, yet their replication is autophagy-independent

**DOI:** 10.1007/s00018-022-04578-7

**Published:** 2022-10-25

**Authors:** Petra Tavčar Verdev, Maja Potokar, Miša Korva, Katarina Resman Rus, Marko Kolenc, Tatjana Avšič Županc, Robert Zorec, Jernej Jorgačevski

**Affiliations:** 1 Laboratory of Neuroendocrinology-Molecular Cell Physiology, Institute of Pathophysiology, Faculty of Medicine, University of Ljubljana, Ljubljana, Slovenia; 2 Celica Biomedical, Ljubljana, Slovenia; 3 Institute of Microbiology and Immunology, Faculty of Medicine, University of Ljubljana, Ljubljana, Slovenia

**Keywords:** Astrocyte, Autolysosome, Autophagosome, Flavivirus, Infection, LC3

## Abstract

Astrocytes, an abundant type of glial cells, are the key cells providing homeostasis in the central nervous system. Due to their susceptibility to infection, combined with high resilience to virus-induced cell death, astrocytes are now considered one of the principal types of cells, responsible for virus retention and dissemination within the brain. Autophagy plays an important role in elimination of intracellular components and in maintaining cellular homeostasis and is also intertwined with the life cycle of viruses. The physiological significance of autophagy in astrocytes, in connection with the life cycle and transmission of viruses, remains poorly investigated. In the present study, we investigated flavivirus-induced modulation of autophagy in human astrocytes by monitoring a tandem fluorescent-tagged LC3 probe (mRFP-EGFP-LC3) with confocal and super-resolution fluorescence microscopy. Astrocytes were infected with tick-borne encephalitis virus (TBEV) or West Nile virus (WNV), both pathogenic flaviviruses, and with mosquito-only flavivirus (MOF), which is considered non-pathogenic. The results revealed that human astrocytes are susceptible to infection with TBEV, WNV and to a much lower extent also to MOF. Infection and replication rates of TBEV and WNV are paralleled by increased rate of autophagy, whereas autophagosome maturation and the size of autophagic compartments are not affected. Modulation of autophagy by rapamycin and wortmannin does not influence TBEV and WNV replication rate, whereas bafilomycin A1 attenuates their replication and infectivity. In human astrocytes infected with MOF, the low infectivity and the lack of efficient replication of this flavivirus are mirrored by the absence of an autophagic response.

**Supplementary Information:**

The online version contains supplementary material available at 10.1007/s00018-022-04578-7.

## Introduction

Autophagy is an evolutionary conserved pathway for lysosome-mediated degradation of intracellular components [1]. Macroautophagy (hereafter referred to as autophagy) is a form of autophagy in which substrates destined for degradation are sequestered in specialized double-membrane compartments termed autophagosomes [1–3]. Various intrinsic and environmental factors trigger autophagy [4] by inducing the formation of a phagophore, which engulfs cytoplasmic components, including proteins, protein aggregates, ribosomes and organelles, all finally ending up in an autophagosome [3, 5]. Then autophagosomes mature by fusing with endosomes and lysosomes forming amphisomes and autolysosomes, respectively [6–8]. Ultimately, cellular cargo is degraded by lysosomal hydrolases in autolysosomes, and recycled nutrients are released back into the cell cytoplasm [1]. In normal conditions, autophagy maintains cellular homeostasis by removing long-lived or damaged proteins and organelles [2, 9]. However, in various stress conditions, including starvation, growth factor deprivation, hypoxia and intracellular pathogen infections, autophagy is induced to supply nutrients from intracellular material and to promote cell survival [1, 10, 11]. Moreover, during various pathogen infections, autophagy is involved in regulating immunity and inflammation to contain the pathogen burden [12].

After infections with pathogenic viruses, host cell autophagy can be exploited and manipulated in different ways. Depending on the virus, host species, cell type and cellular environment, autophagy plays either an anti- or pro-viral role in the virus life cycle and its pathogenesis [13–18]. The antiviral effect of autophagy has been linked to selective virus degradation via xenophagy, a process that involves clearing microorganisms, including viruses, as well as autophagy-mediated pathogen recognition and activation of inflammatory response, leading to elimination of infected cells [12, 13, 15]. However, some viruses have evolved mechanisms to bypass the autophagy-mediated antiviral response or even to modify the autophagic machinery to support virus survival and replication within the host cell [13, 18, 19].

Several members of the genus Flavivirus, which include single-stranded positive-sense RNA viruses transmitted by arthropods (mosquitos and ticks), are severe human pathogens, eliciting millions of infections annually over a wide geographic range [20–22]. Some, including West Nile virus (WNV), tick-borne encephalitis virus (TBEV), Japanese encephalitis virus (JEV) and Zika virus (ZIKV), are considered neurotropic, which reflects their ability to invade the central nervous system (CNS) and infect neural cells [22–24]. They enter the CNS by crossing the blood–brain barrier (BBB) and/or through the peripheral nervous system [22, 23, 25, 26]. In the CNS, they may become neurovirulent (i.e., they replicate and cause damage in the CNS), evoking severe and life-threatening neurological complications, such as encephalitis, meningitis, myelitis and cognitive impairment [19, 20, 22, 27]. These conditions are inevitably linked to multiple responses at the cellular level; flavivirus-mediated infections have been shown to trigger neuronal death, activation of microglia, reactive astrogliosis and astrocyte demise, BBB disruption, and accumulation of leukocytes and macrophages in different brain regions, including the cerebral cortex [20, 21, 23, 26, 28]. Viral infections of astrocytes, the most heterogeneous and abundant glial cells in the CNS [29], have gained a particular interest in recent years, because these cells execute key homeostatic and metabolic functions in the CNS (e.g., they control ionic and water balance, regulate blood flow, uptake and recycle neurotransmitters and are actively involved in synaptic signaling) [30, 31], and are highly susceptible to infections with viruses circulating in the vascular system [22, 24, 28, 32–34]. After a virus crosses the BBB, astrocytes are one of first CNS cell types where virus uptake occurs [22]. Recent reports revealed high susceptibility of astrocytes to infection with ZIKV [34–36] and TBEV [24, 33]. In addition, high resilience to virus-induced cell death was reported for astrocytes after infection with ZIKV, TBEV and WNV [24, 33, 34, 37, 38], indicating that astrocytes might serve as hubs and reservoirs for viral replication, retention and dissemination within the brain [22]. Not all flaviviruses have recognized pathogenic effects in mammals, because the existence of flaviviruses without a known vertebrate host has been reported [39, 40]. Flaviviruses affect various cellular processes in astrocytes. In particular, alterations in organelle morphology, cytoskeleton polymerization and structure, vesicle trafficking, production of inflammatory mediators and reactive oxygen species, were observed in flavivirus-infected astrocytes [24, 33, 41–43]. However, autophagy in astrocytes infected with viruses is poorly explored.

In this study, we infected human astrocytes with two confirmed neurovirulent flaviviruses (TBEV and WNV) and with mosquito-only flavivirus (MOF), which is considered non-pathogenic. We assessed their infectivity, replication rate, and their effect on autophagy, as well as the involvement of autophagy in virus replication. Human astrocytes proved to be susceptible to infection with TBEV, WNV and, surprisingly, also to MOF. Moreover, using a validated fluorescence reporter system to monitor the dynamics of autophagy, we have demonstrated that TBEV and WNV, which successfully infect and replicate in human astrocytes, increase the rate of autophagy. However, the autophagosome maturation process and the size of the autophagic compartments (autophagosomes and autolysosomes) were not affected. In addition, pharmacological modulation of early stages of autophagy had no effect on TBEV and WNV replication. Interestingly, the infection rate of human astrocytes with MOF was found to be significant, albeit very low, in line with the concentration of MOF RNA, which remained low throughout the experiment. MOF also did not affect autophagy dynamics or autophagic compartment size in this cell type. In summary, our results show that pathogenic flaviviruses efficiently trigger the autophagic response in astrocytes. However, replication of flaviviruses is likely an autophagy-independent process in this cell type.

## Materials and methods

### Cell cultures

Cryopreserved primary human astrocytes were purchased from Innoprot (P10251, P10254). After thawing, cells were grown in cell culture flasks for 4 days and then plated on glass coverslips coated with poly-d-lysine (diameter 22 mm; Sigma-Aldrich, P6407) at a cell density of 1.5 × 10^4^ cells per coverslip (transfection and immunocytochemistry experiments) or on 12-well culture plates (TPP, 92112) at a cell density of 3 × 10^4^ cells per well (RT-PCR experiments) as measured by a Scepter cell counter (Merck KGaA, Darmstadt, Germany). Cell cultures were maintained in astrocyte growth medium (high glucose Dulbecco’s modified Eagle’s medium [Sigma-Aldrich, D5671] supplemented with 10% fetal bovine serum [FBS; Sigma-Aldrich, F7524], 1 mM sodium pyruvate [Sigma-Aldrich, S8636], 2 mM l-glutamine [Sigma-Aldrich, G3126], 5 U/ml penicillin, and 5 µg/ml streptomycin [Sigma-Aldrich, P0781]) at 37 °C, 5% CO_2_ atmosphere, and 95% relative humidity. Cells were used in experiments approximately 24 h after plating on coverslips/culture plates.

### Flaviviruses and infection of human astrocytes

Experiments involving viruses were carried out in biosafety level 3 facility. For infection of human astrocytes, the following strains were used: TBEV (TBEV strain Ljubljana 1; deposited in the EVA-GLOBAL Virus Archive under reference number Ref-SKU: 007v-EVA71), WNV (West Nile virus/Slovenia/Ko169/2018; Ref-SKU: 007V-03831), and MOF (isolated from *Aedes albopictus* in Slovenia [44]).

Virus stock solutions were prepared by growing TBEV and WNV in Vero E6 cells in Dulbecco’s modified Eagle’s medium with high glucose and GlutaMAX supplement (Thermo Fisher Scientific, 61965026) supplemented with 4% FBS (Euroclone, ECS0180L) at 37 °C for 7 days. A stock solution of MOF was prepared by growing virus in C6/36 cells (*Aedes albopictus* cell line, kindly provided by Dr. David H. Walker, University of Texas Medical Branch, Department of Pathology, Galveston, Texas, USA) in Leibovitz’s L-15 medium (Thermo Fisher Scientific, 11415064) supplemented with 10% FBS (Euroclone, ECS0180L) at 28 °C. Cell culture supernatants were collected and centrifuged twice at 4 °C (10 min at 3200×*g* and 5 min at 20,800×*g*) in an Eppendorf 5804R centrifuge (Eppendorf, Hamburg, Germany). Supernatants containing viruses were diluted and collected into aliquots.

To mimic conditions that are present during natural infection of cells, the lowest concentration of viruses that yielded reliable infections were used. Infections of human astrocytes performed with all three viruses (TBEV, WNV, and MOF), at the multiplicity of infection (MOI) of 0.1 and of 1, produced similar replication kinetics and infection rates, except of WNV at an MOI of 0.1 where the replication kinetics was minute (data not published). Cultured human astrocytes were inoculated with respective flaviviruses at an MOI of 0.1 (TBEV, MOF) or 1 (WNV), and incubated at 37 °C, 5% CO_2_ atmosphere, and 95% relative humidity.

### Quantitative reverse transcription polymerase chain reaction (RT-PCR)

To monitor the production of flavivirus RNA with RT-qPCR, supernatants were collected at 0, 24, 48, 72, and 96 h post infection (hpi). Using an EZ1 Virus Mini Kit (QIAGEN, 955134) the total nucleic acid was extracted from cell culture supernatants and viral RNA was quantified using RT-qPCR on a QuantStudio 7 Pro Real-Time PCR System (Thermo Fisher Scientific, Waltham, MA, USA). Assays were performed in duplicate on cells from at least two human donors (purchased from Innoprot, see chapter Cell cultures for details).

In experiments where the role of autophagy in flavivirus replication was assessed, cells were first exposed to autophagy modulators (see chapter Autophagy modulation) for 2 h and then inoculated with viruses.

### Measurements of virus infectivity by TCID50 assay

Virus infectivity of progeny virus released from astrocytes was determined by calculating the median tissue culture infectious dose (TCID50). Briefly, Vero E6 cells (ATTC CRL-1586) were seeded at the concentration of 1 × 10^5^/100 µl/cells in 96-well plates (TPP, 92196), supplemented with the growth medium consisting of DMEM with GlutaMAX supplement (Thermo Fisher Scientific, 61965026) and 10% FBS (Euroclone, ECS0180L) and incubated for 24 h at 37 °C in 5% CO_2_. Then, 50 µl of the media was removed from the cells and supplemented with tenfold serial dilutions of suspension collected from the supernatant of virus-infected human astrocytes in 8 replicates. Plates were further incubated at 37 °C with 5% CO_2_. After 6 days plates were inactivated with 100 µl of 4% formaldehyde solution for 30 min. Inactivated plates were visually inspected under the microscope for the presence of cytopathic effect. TCID50 was calculated by the Spearman & Kärber algorithm [45].

### Immunocytochemistry

Immunocytochemical labeling was performed as described previously [33]. Briefly, cells were washed with phosphate-buffered saline (PBS; Sigma-Aldrich, P4417) and fixed in 4% formaldehyde solution (in PBS) for 15 min at room temperature. Cells were then permeabilized with Triton X-100 (Merck Millipore, 1086031000) for 10 min at room temperature. After fixation and permeabilization, cells were washed with PBS and incubated in 10% goat serum (Sigma-Aldrich, G6767) and 3% bovine serum albumin (Sigma-Aldrich, A2153) solution in PBS for 1 h at 37 °C to reduce non-specific immunostaining. After washing with PBS, cells were incubated with primary antibodies against flavivirus group antigen (Sigma-Aldrich, MAB10216; dilution 1:600), early endosome antigen 1 (EEA1; Abcam, ab2900; dilution 1:1000) or p62/SQSTM1 (Abcam, ab109012; dilution 1:800), for 2 h at 37 °C or overnight at 4 °C. When double immunostaining was performed, samples were incubated with the first primary antibodies for 2 h at 37 °C, washed with PBS, and then incubated with the second primary antibodies overnight at 4 °C. Afterward, cells were washed with PBS and secondary antibodies against mouse or rabbit IgGs coupled to Alexa Fluor 546 (Invitrogen, Thermo Fisher Scientific, A11003; dilution 1:600) and/or Alexa Fluor 488 (Invitrogen, Thermo Fisher Scientific, A11008; dilution 1:600), respectively, were applied for 45 min at 37 °C, protected from light (the mixture of secondary antibodies was applied simultaneously). To diminish fluorescence bleaching and to stain nuclei, SlowFade Gold Antifade Mountant with 4′,6-diamidino-2-phenylindole (DAPI; Thermo Fisher Scientific, S36942) was used. Control samples (incubated with secondary antibodies alone) were prepared in parallel to determine non-specific staining.

### Plasmids and transfection

To visualize autophagic compartments (autophagosomes and autolysosomes), cells were transfected with plasmid ptfLC3, which encodes fusion protein mRFP-EGFP-LC3 (Addgene, plasmid #21,074 [46]) for 48 h. The transfection protocol was performed with FuGENE 6 reagent (Promega Corporation, E2691) according to the manufacturer’s instructions. In experiments designed to monitor the effect of flaviviruses on autophagy, cells were transfected for 48 h and inoculated with respective viruses 12, 24 or 48 h prior to the fixation, which terminated the experiment. Autophagy modulators (when applicable) were added to the growth medium, which was used in the last step of the transfection protocol (cells were maintained in the same medium for the total duration of 48 h).

### Autophagy modulation

For induction or inhibition of autophagy, the following reagents were used: Earle’s balanced salt solution (EBSS; Sigma-Aldrich, E6267), 1 μM rapamycin (Rapa; Sigma-Aldrich, FL37094), 100 nM wortmannin (Wort; Cayman Chemical Company, 10010591), and 100 nM bafilomycin A1 (Baf; Santa Cruz Biotechnology, sc201550). Rapa, Wort, and Baf stock solutions were prepared in dimethyl sulfoxide (Sigma-Aldrich, D2438) and stored at − 20 °C. Working solutions were prepared in astrocyte growth medium immediately before the experiments.

After transfection with ptfLC3 (for 48), human astrocytes were either exposed to starvation conditions induced by EBSS or treated with pharmacological autophagy modulators for 3 h. In experiments where a combination of modulators was used, cells were first exposed only to Wort/Baf for 30 min and then to a solution containing Wort/Baf and Rapa for additional 3 h. Control astrocytes were maintained in astrocyte growth medium. During treatment, cells were maintained at 37 °C, 5% CO_2_ atmosphere, and 95% relative humidity. After 3 h, cells were fixed in 4% formaldehyde solution (in PBS) for 15 min at room temperature, mounted onto glass slides using SlowFade Gold Antifade Mountant (Thermo Fisher Scientific, S36940). In experiments involving infection with flaviviruses, pharmacological modulators were added either 2 h before the inoculation with viruses (measurements of viral replication, infectious virus production and percentages of infected cells) or in the last step of ptfLC3-transfection protocol, which was shortly followed by virus inoculation (measurements of autophagy in flavivirus-infected astrocytes).

### Fluorescence microscopy

After exposing cells to starvation conditions or autophagy modulators for 3 h (experiments for validation of the ptfLC3 autophagy reporter), mRFP-EGFP-LC3-expressing cells were imaged by SIM (ELYRA PS.1, Zeiss, Oberkochen, Germany) using an oil-immersion objective (Plan-Apochromat 63 × /1.4 Oil DIC M27, Zeiss). Individual cells were imaged by obtaining multiple 500-nm-thick *z* stacks (3–7 stacks per cell). EGFP and mRFP fluorescence was excited with 488 and 561 nm laser beams, respectively. The emitted fluorescence was collected through bandpass emission filters (495–575 nm [EGFP] and 570–650 nm [mRFP]) and detected with an EMCCD camera (iXon 885, Andor, Oxford Instruments, Abingdon, UK). Imaging settings were determined based on the fluorescence intensity profiles of control samples and maintained unchanged for imaging of all samples.

In the experiments, where we investigated flavivirus infection rates and flavivirus-induced modulation of autophagy, imaging was performed with confocal microscopes LSM780 and LSM800 (Zeiss, Oberkochen, Germany). To determine the percentage of flavivirus-infected cells and to monitor autophagy after flavivirus infection, we used LSM800, a dry objective (20 × /0.8 M27, Zeiss) and an oil-immersion objective (63 × /1.4 Oil DIC M27, Zeiss), respectively. Alexa Fluor 564 dye was excited with the 561 nm diode laser and the emission was measured at 560–700 nm. DAPI was excited with the 405 nm diode laser and the emission at 400–530 nm was detected. EGFP and mRFP fluorescence was excited with 488 and 561 nm laser beams, respectively. Emitted fluorescence was measured at 410–560 nm (EGFP) and 560–654 nm (mRFP), respectively. As described above, multiple stacks (500 nm thick) were imaged per individual cell expressing mRFP-EGFP-LC3. Imaging of p62/SQSTM1 as well as of the colocalization between EEA1 and TBEV or WNV was performed with LSM780, using an oil immersion objective (63 × /NA 1.4, Zeiss). Fluorescence of p62/SQSTM1 and EEA1, immuno-labeled with Alexa Fluor 488, was excited by argon laser (488 nm), and the emission light was collected through a band-pass filter (505–530 nm). Fluorescence of TBEV and WNV, immuno-labeled with Alexa Fluor 546, was excited with the He/Ne laser (546 nm), and the emission light was filtered with a long-pass filter with a cut-off below 560 nm.

### Transmission electron microscopy (TEM)

Astrocytes were grown on translucent Corning polyethylene terephthalate Transwell permeable supports (Merck, Darmstadt, Germany). Controls and Rapa-treated cells were fixed using modified Karnovsky fixative. After post-fixation with 1% OsO_4_ and dehydration in a series of ethanol solutions, astrocytes were embedded in EPON resin (Serva, Heidelberg, Germany). Ultrathin sections were contrasted with 3% lead citrate and 0.5% uranyl acetate using an automatic contrasting system (EM AC 20; Leica, Wetzlar, Germany). Sample imaging was performed at 120 kV using TEM JEM-1400 Plus (JEOL, Tokyo, Japan) and Ruby CCD camera (JEOL, Tokyo, Japan).

### Image analysis

#### Autophagy dynamics

The number of fluorescently labeled objects in a single cell expressing mRFP-EGFP-LC3 was determined with ImageJ software (JACoP plugin [47]). We determined the number of co-localized objects (mRFP^+^EGFP^+^) and red-only objects (mRFP^+^EGFP^−^), which correspond to autophagosomes and autolysosomes, respectively. Objects in all imaged z stacks (in the whole cell) were counted. The total number of autophagic compartments was calculated as a sum of autophagosomes and autolysosomes and was normalized to a unit volume (1000 μm^3^) to compensate for the variability in cell sizes. The ratio of autolysosomes to autophagosomes was determined to assess potential differences in autophagosome maturation between control cells and cells treated with autophagy modulators or infected with flaviviruses. We calculated the estimated volume of each cell by multiplying the cell area (determined by delineating individual cells imaged under the transmission light) with the height of a cell (the number of imaged z stacks multiplied by 500 nm, which represents the thickness of an individual z stack).

The density, the volume and the fluorescence intensity of immuno-labeled p62/SQSTM1 particles were determined in all imaged z stacks (in the whole cell) with ImageJ software (3D Objects Counter plugin [47]).

#### Size of autophagic compartments

The diameter of individual autophagic compartments (autophagosomes and autolysosomes) was determined in mRFP-EGFP-LC3-expressing cells, imaged by fluorescence microscopy (SIM and confocal microscopy). Specifically, the full width at half maximum of the red fluorescence (mRFP) signal intensity was measured using ZEN microscope software (Zeiss, Oberkochen, Germany). All vesicle diameter values represent an average of two independent measurements made in the equatorial plane in two perpendicular directions (horizontally and vertically) to improve the accuracy of the measurement. We measured the diameter of all autophagic compartments that were in focus in the equatorial *z* stack of individual imaged cells.

The size of the autophagic compartments was also determined on TEM micrographs. Autophagy-related structures were identified based on specific morphological characteristics, such as spherical structures with a double-layered membrane containing different intercellular material, and their diameter was measured using SightX Viewer software (JEOL, Tokyo, Japan).

#### Infection rate, viral replication and colocalization between viral particles and early endosomes

To determine the infection rates of different flaviviruses, cells were immuno-labeled with antibodies against flavivirus group antigen and stained with DAPI. We counted the flavivirus group antigen-positive cells and the number of DAPI-stained nuclei in multiple fields of view. The percentage of infected cells was calculated as a ratio between the number of flavivirus group antigen-positive cells and the number of all cells in individual fields of view. In addition, virus replication dynamics (in control conditions and after exposure to pharmacological autophagy modulators) was assessed by measuring the concentration of viral RNA in the supernatant of infected cell cultures at different time points after infection. Data were normalized to initial values (obtained at 0 hpi time point) of each sample. The production of infectious viral particles was determined by TCID50 assay. The colocalization between viral particles (TBEV or WNV) and early endosomes was measured in all imaged *z* stacks (in the whole cell) with ImageJ software (JACoP plugin [47]).

### Statistical analysis

All experiments were performed at least in a duplicate, in one (exposure to starvation conditions, flavivirus infection experiments with and without autophagy modulators), or two (exposure to pharmacological autophagy modulators for validation of the ptfLC3-based autophagy reporter, viral RNA and TCID50 measurements) independent repetitions. We used the Mann–Whitney *U* test (SigmaPlot, Systate Software Inc) to assess statistical differences between two groups of data (*P < 0.05, **P < 0.01, ***P < 0.001) or one-way ANOVA followed by Dunn’s test or Dunnett’s test (SigmaPlot, Systate Software Inc) for comparison of multiple groups (*P < 0.05 was considered statistically significant). Data are presented as boxplots (full lines represent median values and dotted lines represent average values), bar plots (average ± standard error) or logarithmic scale graphs (single time point is presented as the average ± standard error).

## Results

**Fig. 1:**
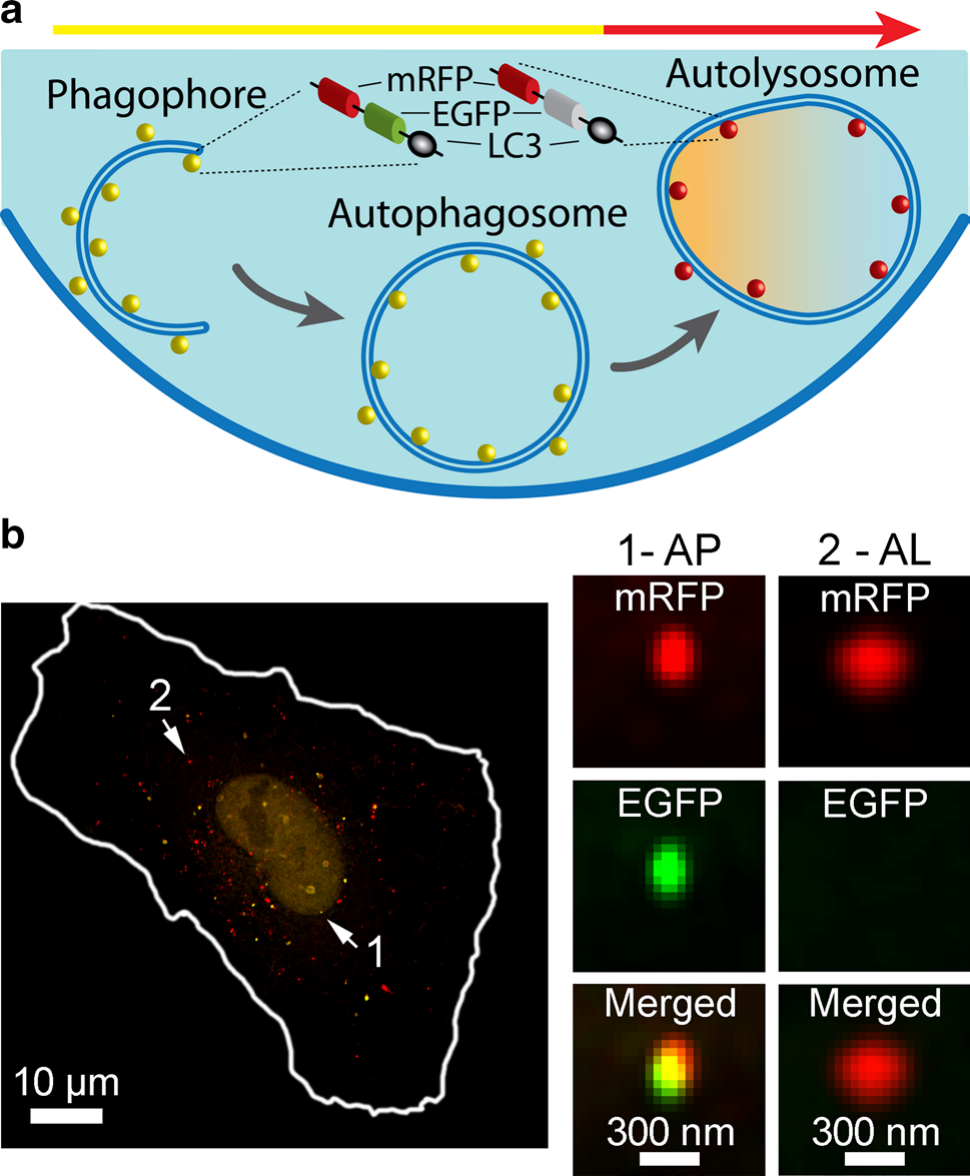
Expression of fusion protein mRFP-EGFP-LC3 as a tool for monitoring autophagosomes and autolysosomes in human astrocytes. **a** Schematic representation of the method for monitoring autophagy by fluorescence microscopy. The panel highlights a cell transfected with plasmid ptfLC3 that encodes mRFP-EGFP-LC3, where LC3, a marker of autophagic compartments, is tandem fluorescent-tagged with mRFP (red fluorescence) and EGFP (green fluorescence). The pH of autophagosomes (AP) is close to neutral, which facilitates the fluorescence of both fluorophores, resulting in yellow objects. Fusion of AP with lysosomes yields autolysosomes (AL), i.e., organelles with acidic pH, where the EGFP fluorescence is quenched, resulting in red-only objects. Fluorescence imaging of ptfLC3-transfected cells allows quantification of AP and AL, and hence can be used to evaluate the extent of autophagosome formation and maturation in single cells. **b** Human astrocyte expressing mRFP-EGFP-LC3, imaged by structured illumination microscopy. Arrows 1 and 2 point to two fluorescent objects magnified in panels adjacent to the larger micrograph. Object 1 represents an AP, as it exhibits red and green fluorescence (mRFP^+^EGFP^+^), and object 2 can be identified as an AL, considering that only the red fluorescence is present (mRFP^+^EGFP^−^). The cell shape is outlined with the white line

To learn whether flavivirus infection affects autophagy in human astrocytes, we first validated the methodological approach to evaluate the dynamics of autophagy. We transfected human astrocytes with the plasmid ptfLC3, which encodes tandem fluorescent-tagged LC3 (microtubule associated protein 1 light chain 3), a marker protein integrated in the inner and outer membranes of autophagic structures [48–50]. This fusion protein consists of LC3 and two fluorescent tags: monomeric red fluorescent protein (mRFP) and enhanced green fluorescent protein (EGFP) (mRFP-EGFP-LC3; Fig. 1a) [46]. The difference in the p*K*a values of these two fluorophores (4.5 for mRFP and 6 for EGFP [51, 52]), and consequently their pH sensitivity, was exploited to differentiate between neutral and acidic autophagy intermediates. In the neutral environment of autophagosomes, mRFP and EGFP are both stable and emit fluorescence (observed as a yellow signal; Fig. 1a). However, after the transition from autophagosomes to autolysosomes, the luminal environment becomes more acidic, leading to EGFP fluorescence attenuation, whereas mRFP fluorescence remains bright (observed as red-only puncta; Fig. 1a) [46]. Hence, in fluorescence micrographs, autophagosomes are recognized as dually labeled mRFP^+^/EGFP^+^ objects, and autolysosomes are observed as mRFP^+^/EGFP^−^ puncta (Fig. 1b).

### Autophagy dynamics is affected by starvation and pharmacological modulators in human astrocytes

**Fig. 2:**
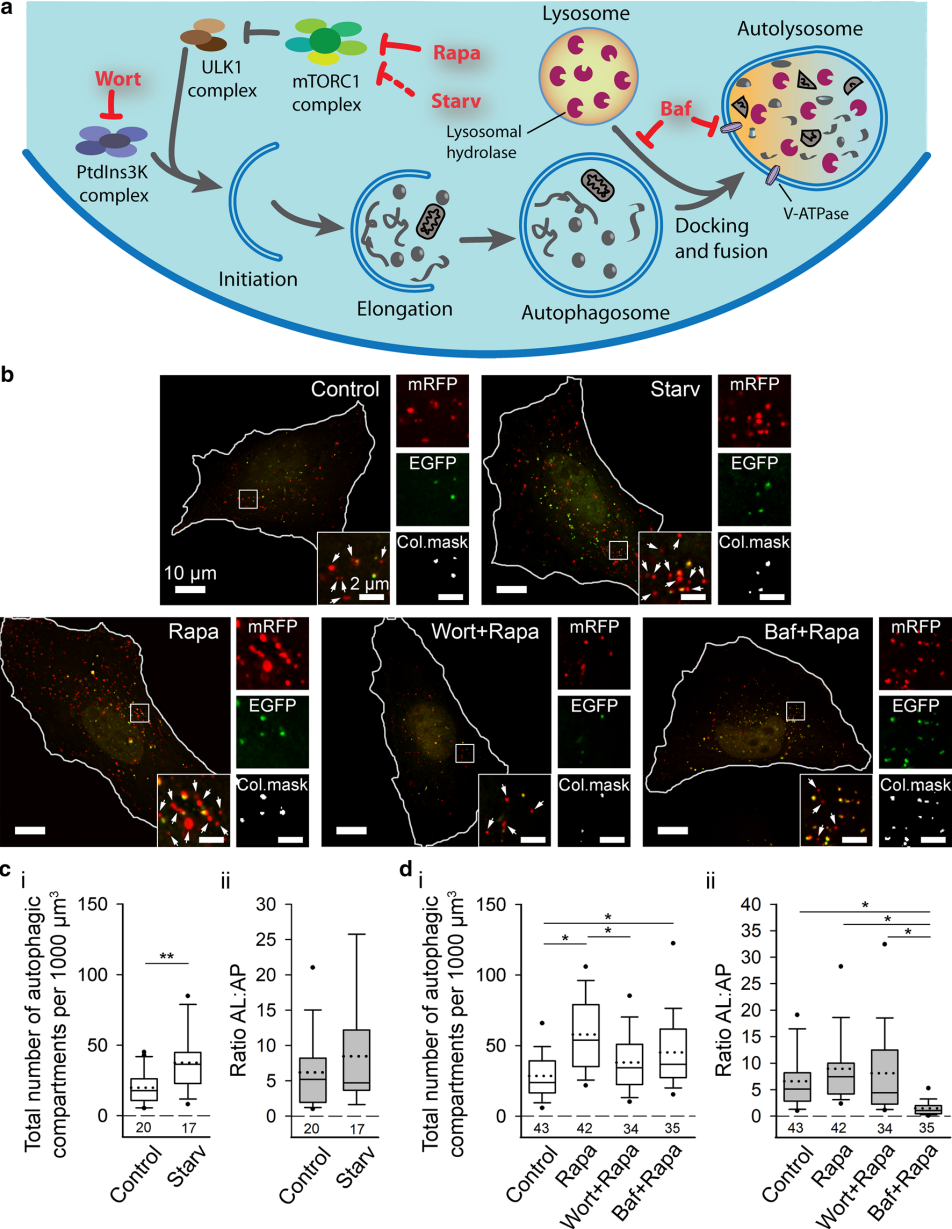
Starvation and pharmacological autophagy modulators affect autophagy dynamics in human astrocytes. **a** Schematic representation of the autophagic pathway showing the proposed action of selected autophagy modulators. Inhibition of mammalian target of rapamycin complex 1 (mTORC1) complex by rapamycin (Rapa) or starvation conditions (Starv) increases autophagy [4, 53, 55]. Wortmannin (Wort) suppresses the early stages of autophagy by inhibiting class III phosphoinositide 3-kinase (PtdIns3K) complex [56]. Bafilomycin A1 (Baf) inhibits the late stages of autophagy by preventing fusion of autophagosomes (AP) with lysosomes and by impeding autolysosome (AL) acidification (inhibition of V-ATPase proton pumps) [59]. **b** Representative structured illumination micrographs of human astrocytes expressing mRFP-EGFP-LC3 in control conditions (Control), nutrient and serum deprivation conditions (Starv), and after exposure to selected autophagy modulators (Rapa, Wort + Rapa, Baf + Rapa). White rectangles delineate a cell area, which is magnified in four smaller panels adjacent to the image of the cell, showing mRFP fluorescence (mRFP), EGFP fluorescence (EGFP), colocalization of both signals (Col.mask), and an overlay of both signals. White arrows in the panels showing an overlay of mRFP and EGFP fluorescence indicate AL (red-only objects), and colocalized signals (Col.mask) represent AP. White outlines delineate cell shapes. **c** Starv induces an increase in the total number of autophagic compartments (**c**_**i**_, **P < 0.01, Mann–Whitney *U* test) without affecting the ratio AL:AP (**c**_**ii**_, *P* > 0.05, Mann–Whitney *U* test) versus control conditions. **d** Rapa and Baf + Rapa, but not Wort + Rapa, increase the total number of autophagic compartments compared with controls (**d**_**i**_, *P < 0.05, one-way ANOVA followed by Dunn’s test). Treatment with Wort + Rapa results in a lower number of autophagic compartments versus Rapa (**d**_**i**_, *P < 0.05, one-way ANOVA followed by Dunn’s test). Conversely, of all treatments, only Baf + Rapa significantly decreases the ratio AL:AP (**d**_**ii**_, *P < 0.05, one-way ANOVA followed by Dunn’s test). Full lines in the boxplots represent median values and dotted lines correspond to average values. The numbers below the boxplots are the number of cells analyzed for each condition. ULK1, Unc-51-like autophagy activating kinase 1

To validate the use of plasmid ptfLC3 for measurement of autophagy dynamics, human astrocytes were exposed to either starvation (incubation in EBSS, devoid of nutrients and growth factors) or to pharmacological autophagy modulators (1 μM Rapa, 100 nM Wort, 100 nM Baf). These autophagy modulators have been shown to either induce or impede the course of autophagy (Fig. 2a). Deprivation of nutrients and/or growth factors was shown to stimulate autophagic activity by interacting with various signaling pathways, ultimately leading to inhibition of the mammalian target of rapamycin complex 1 (mTORC1) [53]. mTORC1 is a key signaling hub coordinating nutrient status and cell growth, crucially involved in autophagy regulation [4, 54]. In addition, starvation can also induce autophagy in an mTORC1-independent fashion [4]. Rapa induces autophagy by forming a complex with FK506-binding protein (FKBP12) and allosterically inhibiting the mTORC1 [55]. Wort is an inhibitor of phosphoinositide 3-kinases (PtdIns3K) [56], including class III PtdIns3K, i.e., a component of the complex involved in phagophore nucleation [57], and thus inhibits the early stages of autophagy. On the other hand, Baf inhibits autophagy at late stages by inhibiting V-ATPase proton pumps in autolysosomes, thus blocking their acidification [58, 59], and/or by preventing the fusion of autophagosomes with lysosomes [59].

After exposure to starvation conditions or selected autophagy modulators, human astrocytes expressing mRFP-EGFP-LC3 were imaged by SIM (Fig. 2b) and the autophagy rate was assessed based on measurements of the total number of autophagic compartments (calculated as the sum of autophagosomes and autolysosomes) per cell volume and the ratio of autolysosomes to autophagosomes (Fig. 2c, d). Fluctuations in the overall number of LC3-positive puncta are an indicator of autophagic activity, with higher numbers normally representing increased autophagy [50]. However, an increase in the amount of LC3-positive puncta can also be a result of accumulation of autophagosomes due to blockage of events downstream of autophagosome formation (e.g., fusion of autophagosomes with lysosomes and autolysosomal acidification) [49, 50]. To distinguish between these two scenarios, we also assessed the ratio of autolysosomes to autophagosomes. A higher number of LC3-positive compartments combined with an unchanged or even increased ratio of autolysosomes to autophagosomes correspond to increased rate of functional autophagy. In contrast, a decreased ratio of autolysosomes to autophagosomes is associated with blockage of late autophagy steps, such as diminished autophagosome maturation due to inhibited fusion between autophagosomes and lysosomes.

Starvation, which affects the early stages of autophagy (Fig. 2a), increased autophagy in human astrocytes. Specifically, in cells maintained in a nutrient-deprived environment, the total number of autophagic compartments per unit volume was ~ twofold higher compared with controls maintained in normal growth conditions (38 ± 5 versus 20 ± 3 autophagic objects per 1000 μm^3^; Fig. 2c_i_; **P < 0.01, Mann–Whitney *U* test). Conversely, the ratio of autolysosomes to autophagosomes remained unchanged (Fig. 2c_ii_; *P* > 0.05, Mann–Whitney *U* test), indicating that in human astrocytes, starvation enhances autophagy without affecting the transition rate between early and late intermediates of this pathway. Similarly, treatment of human astrocytes with Rapa also stimulated autophagic activity, because we observed a ~ twofold increase in the total number of autophagic compartments (58 ± 4 versus 28 ± 3 autophagic objects per 1000 μm^3^; Fig. 2d_i_; *P < 0.05, one-way ANOVA followed by Dunn’s test) and an unchanged ratio of autolysosomes to autophagosomes (Fig. 2d_ii_; *P* > 0.05, one-way ANOVA). In agreement with the established mechanism of the inhibitory action of Wort (Fig. 2a), treatment of human astrocytes with Wort prevented the induction of autophagy by Rapa. Specifically, in cells co-treated with Wort and Rapa, the total number of autophagic compartments was ~ 1.5-fold lower compared with cells treated with Rapa alone (38 ± 4 versus 58 ± 4 autophagic objects per 1000 μm^3^) and comparable with untreated control cells (Fig. 2d_i_; *P < 0.05, one-way ANOVA followed by Dunn’s test). In contrast, human astrocytes co-treated with Baf and Rapa did not differ in the total number of autophagic compartments (Fig. 2d_i_; *P* > 0.05, one-way ANOVA), but exhibited a ~ sixfold decrease in the ratio of autolysosomes to autophagosomes compared with cells treated with Rapa alone (1.5 ± 0.2 versus 8.9 ± 1.1; Fig. 2d_ii_; *P < 0.05, one-way ANOVA followed by Dunn’s test), indicating that Baf blocks autophagosome maturation and the formation of functional autolysosomes in human astrocytes. Similar results were obtained also in rat astrocytes (Fig. S1a).

### The diameter of autophagic compartments differs in normal and stimulated conditions in human astrocytes

**Fig. 3:**
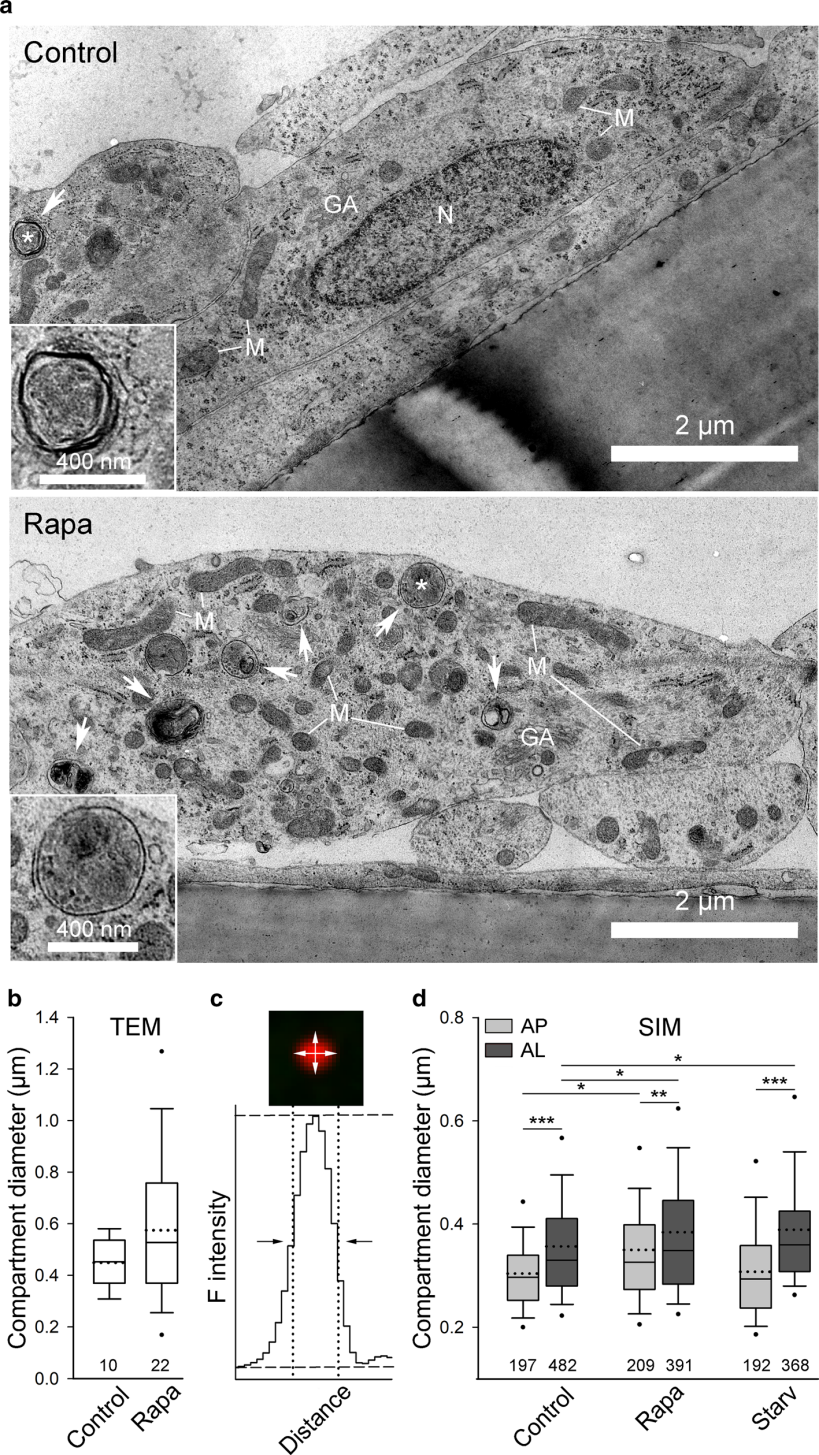
The size of the autophagic compartments differs in normal and stimulated conditions in human astrocytes. **a** Representative transmission electron micrographs of non-treated (Control) and rapamycin-treated (Rapa) human astrocytes. White arrows indicate autophagy-related structures. A single autophagic specimen (marked with a white asterisk) is enlarged in the bottom left corner of the larger micrograph. *GA* Golgi apparatus, *M* mitochondria, *N* nucleus. **b** Ultrastructural analysis of human astrocytes reveals that the diameter of the autophagic compartments is similar between controls and Rapa-treated cells (*P* > 0.05, Mann–Whitney *U* test), although a trend of larger structures was observed after Rapa treatment. **c** Diameter of autophagic compartments in mRFP-EGFP-LC3-expressing human astrocytes, imaged by structured illumination microscopy, was determined by measuring the full width at half maximum of the red fluorescence intensity (F intensity) along the equatorial line in the horizontal and vertical directions. **d** Rapa induces an increase in the diameter of autophagosomes (AP) and autolysosomes (AL) in human astrocytes compared with controls (*P < 0.05, one-way ANOVA followed by Dunn’s test). Alternatively, in starved (Starv) human astrocytes, only AL, but not AP, are larger compared with controls (*P < 0.05, one-way ANOVA followed by Dunn’s test). In all conditions tested, AL are significantly larger compared with AP (***P < 0.001, **P < 0.01, Mann–Whitney *U* test). Full lines in the boxplots represent median values and the dotted lines correspond to average values. Numbers below the boxplots are the number of compartments analyzed in 5 control and 10 Rapa-treated cells (**b**) or in 16 control, 12 starved, and 10 Rapa-treated cells (**d**)

To measure the size of autophagic compartments in human astrocytes, we first used TEM (Fig. 3a). There have been reports that autophagic compartment size differs in normal and stimulated conditions, therefore we measured the diameter of the autophagic compartments in control and Rapa-treated cells. The diameter of identified autophagosomes and autolysosomes was in the range of 305–584 nm in control conditions, and 155–1300 nm in cells treated with Rapa (Fig. 3b). Moreover, we observed a trend of larger autophagic compartments in Rapa-treated cells compared with controls (the average diameter was 575 nm ± 62 nm [*n* = 22] versus 449 nm ± 29 nm [*n* = 10]), although the difference was not statistically significant (Fig. 3b; *P* > 0.05, Mann–Whitney *U* test).

To further investigate possible differences in the size of autophagic compartments between normal and stimulated conditions, we next determined the diameter of autophagosomes and autolysosomes expressing mRFP-EGFP-LC3 using SIM. SIM achieves high spatial resolution (up to 100 nm in the lateral plane) [60]. Therefore, this technique enables accurate size measurements of fluorescently labeled cellular compartments, including vesicular structures that are larger than 100 nm [61, 62]. The diameter of individual vesicles was determined as depicted in Fig. 3c. In normal conditions, the diameter of autophagosomes ranged from 157 to 749 nm, with an average value of 304 nm ± 6 nm (*n* = 197), whereas autolysosomes were significantly larger with a diameter in the range of 189–1545 nm and an average value of 355 nm ± 6 nm (*n* = 482) (Fig. 3d; ***P < 0.001, Mann–Whitney *U* test). Only ~ 4.5% of autophagosomes and ~ 0.5% of autolysosomes in control resting conditions had a diameter less than 200 nm.

We next investigated whether Rapa and starvation conditions affect the diameter of autophagosomes and autolysosomes in human astrocytes (Fig. 3d). In Rapa-treated cells, the average diameter of autophagosomes and autolysosomes was 350 nm ± 8 nm (*n* = 209), and 384 nm ± 7 nm (*n* = 391), respectively. This indicates that Rapa significantly increases the diameter of both autophagic structures (*P < 0.05, one-way ANOVA followed by Dunn’s test). A similar trend was observed in cells exposed to starvation conditions; the average diameter of autolysosomes (389 nm ± 6 nm, *n* = 368) was ~ 10% larger compared with controls (*P < 0.05, one-way ANOVA followed by Dunn’s test). Interestingly, starvation conditions did not affect the average diameter of autophagosomes (308 nm ± 7 nm, *n* = 192) (*P* > 0.05, one-way ANOVA). In cells challenged by Rapa and starvation conditions, autolysosomes were significantly larger than autophagosomes (Fig. 3d; ***P < 0.001, **P < 0.01, Mann–Whitney *U* test), as observed in control cells.

### TBEV and WNV infect and successfully replicate in human astrocytes, and MOF displays low infectivity

**Fig. 4:**
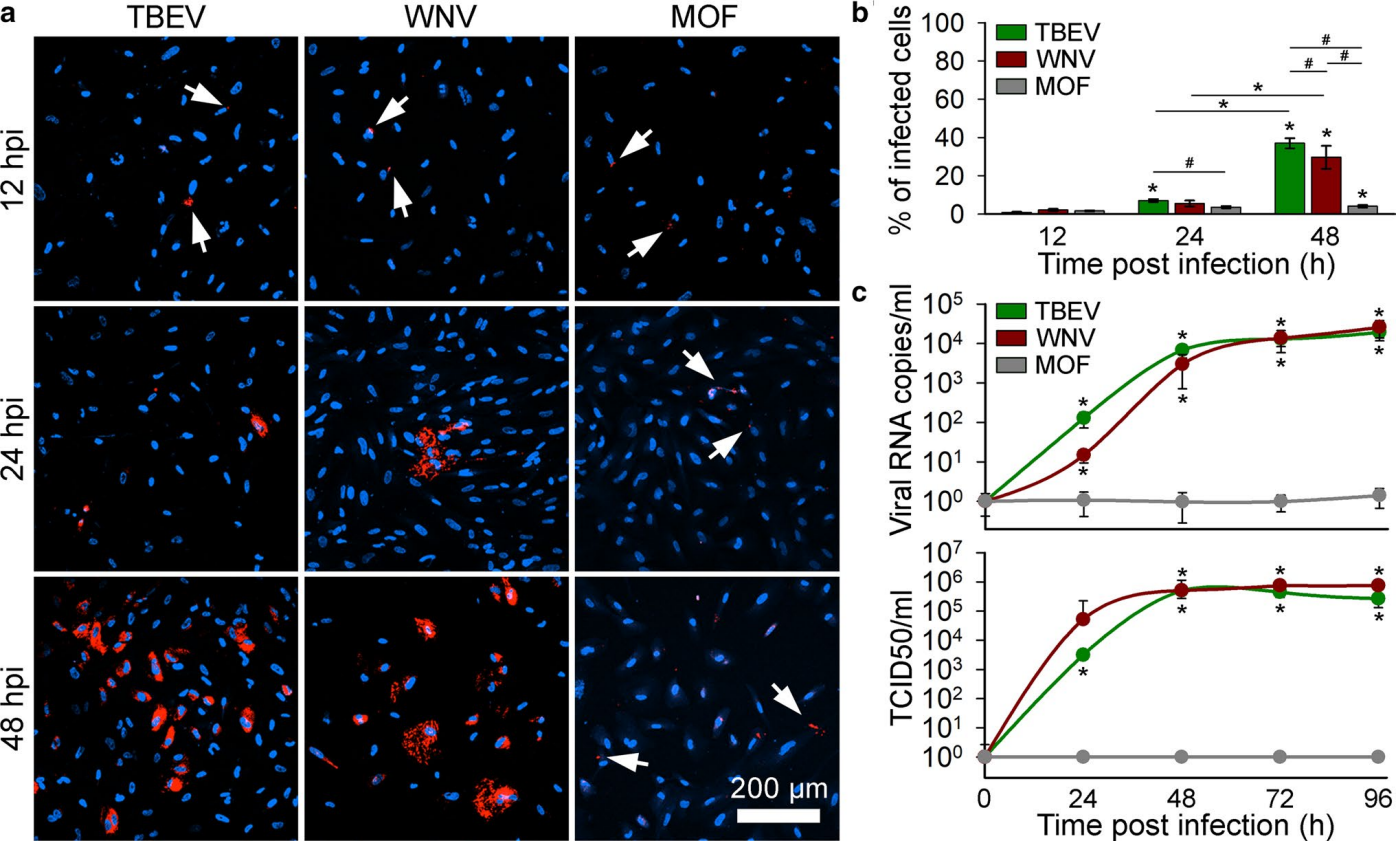
Tick-borne encephalitis virus (TBEV), West Nile virus (WNV), and mosquito-only flavivirus (MOF) infect human astrocytes, but only TBEV and WNV replicate efficiently in human astrocytes. **a** Human astrocytes infected with TBEV, WNV, and MOF and immuno-labeled with antibodies against flavivirus group antigen (red) at 12, 24, and 48 h post infection (hpi). Cell nuclei are stained with 4′,6-diamidino-2-phenylindole (DAPI; blue). Arrows indicate weakly infected cells. **b** The percentages of TBEV-, WNV- and MOF-infected cells at 12, 24, and 48 hpi were determined as the ratio between the number of flavivirus group antigen-positive cells and the number of DAPI-stained nuclei. Infection rates in human astrocytes increase with time for all three flaviviruses (*P < 0.05, one-way ANOVA followed by Dunn’s test). TBEV infection yields the highest percentage of infected cells at 48 hpi compared with WNV and MOF (^#^*P* < 0.05, one-way ANOVA followed by Dunn’s test). Data are presented as an average ± standard error. Statistical comparison was made between the groups of cells infected with the same virus at different hpi (*P < 0.05, one-way ANOVA followed by Dunn’s test) and between samples infected with different viruses at the same infection duration (^#^*P* < 0.05, one-way ANOVA followed by Dunn’s test). Number of cells analyzed at 12, 24, and 48 hpi: TBEV (1363, 1169 and 1550), WNV (674, 1047 and 1081), MOF (1010, 1064 and 773). Number of fields of view analyzed at 12, 24, and 48 hpi: TBEV (20, 20 and 20), WNV (19, 21 and 24), MOF (22, 22 and 21). **c** Replication rates of TBEV, WNV, and MOF in human astrocytes, determined by quantifying the amount of viral RNA in the cell culture supernatant (Viral RNA copies/ml; top graph) and the median tissue culture infectious dose (TCID50/ml; bottom graph) at different hpi (from 0 to 96). The concentration of TBEV and WNV RNA increases with time post infection, reaching the plateau at 48 hpi for both viruses (*P < 0.05, Mann–Whitney *U* test), whereas the quantity of MOF RNA does not change with time post infection in human astrocytes (*P* > 0.05, Mann–Whitney *U* test). TCID50 of TBEV and WNV confirms infectivity of astrocyte-released virus in Vero E6 cells and the concentration of infectious TBEV and WNV increases with time post infection (*P < 0.05, Mann–Whitney *U* test), while released MOF is not infective (*P* > 0.05, Mann–Whitney *U* test). Data are presented on a logarithmic scale as an average ± standard error. Statistical comparison was made against initial values obtained at 0 time point. Cells were infected with TBEV and MOF at an MOI 0.1 and with WNV at an MOI 1

To determine the infection rates of different flaviviruses in human astrocytes, we infected cells with TBEV, WNV, or MOF. At different time points post infection (12, 24, and 48 h) cells were fixed, immuno-labeled with antibodies against flavivirus group antigen (Fig. 4a), and the percentage of infected cells was determined (Fig. 4b). Generally, the infection rates of all flaviviruses increased with time post infection. Specifically, the percentages of infected cells at 12 hpi were low for all flaviviruses tested (0.8% ± 0.3%, *n* = 1363 for TBEV; 2.1% ± 0.6%, n = 674 for WNV; 1.6% ± 0.3%, *n* = 1010 for MOF). At 24 hpi, only the percentage of TBEV-infected cells increased significantly compared with the 12 hpi time point (*P < 0.05, one-way ANOVA followed by Dunn’s test). In addition, we observed significant differences between the percentages of infected cells at 12 hpi and 48 hpi for TBEV, WNV, and also MOF (*P < 0.05, one-way ANOVA followed by Dunn’s test). At 48 hpi, significant differences in the infection rates were observed between all three flaviviruses (^#^*P* < 0.05, one-way ANOVA followed by Dunn’s test); TBEV reached the highest percentage of infected cells (37.0% ± 2.7%, *n* = 1550), followed by WNV (29.6% ± 6.0%, *n* = 1081) and MOF (4.0% ± 0.7%, *n* = 773). These results indicate that TBEV and WNV are more efficient in infecting human astrocytes than MOF.

In parallel, we assessed the rates of viral replication and infectious virus production in human astrocytes by measuring the number of viral RNA copies in the cell culture supernatants and the TCID50 at different time points post infection (0, 24, 48, 72, and 96 h) for all three flaviviruses (Fig. 4c).

A time-dependent increase in viral RNA concentration, compared with 0 hpi, was detected for TBEV and WNV (*P < 0.05, Mann–Whitney *U* test) reaching a plateau for both viruses at ~ 48 hpi, while the concentration of MOF RNA remained unchanged (Fig. 4c, top graph). Likewise, a similar time-dependent enhancement in the production of infectious astrocyte-released TBEV and WNV in comparison to 0 hpi (*P < 0.05, Mann–Whitney *U* test) was detected by TCID50 assay (Fig. 4c, bottom graph). The increase in infectious virus concentration was in the case of TBEV observed already at 24 hpi. For WNV, a statistically significant increase in infectious virus production was observed slightly latter, at 48 hpi. However, both, the RNA amplification dynamics and the production of infectious viruses were similar between TBEV and WNV (*P* > 0.05, one-way ANOVA). Human astrocyte failed to produce infectious MOF (Fig. 4c, bottom graph).

In summary, our results show that TBEV and WNV successfully infect human astrocytes, replicate in them, and lead to the production of infectious viral particles. Moreover, we show that MOF can also infect human astrocytes, albeit at a much lower level compared with the other two flaviviruses tested, and with absent production of infectious virus. The amount of MOF RNA detected in supernatant of infected astrocytes and the concentration of infectious MOF does not change significantly with time, which renders replication of MOF in human astrocytes uncertain.

### Infection of human astrocytes with TBEV and WNV, but not MOF, stimulates autophagy without affecting the autophagosome maturation process

**Fig. 5:**
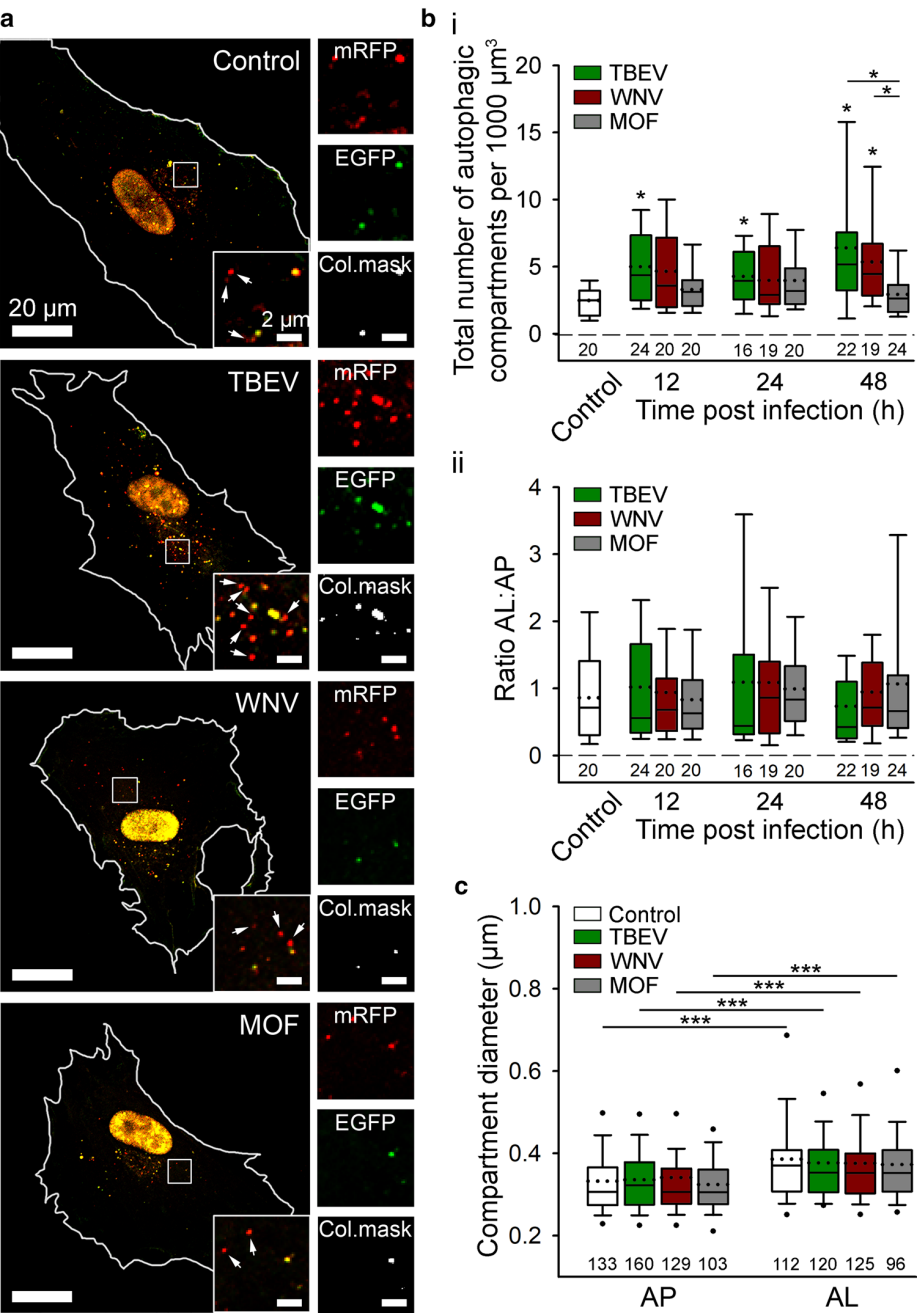
Tick-borne encephalitis virus (TBEV), West Nile virus (WNV), but not mosquito-only flavivirus (MOF), increase autophagy in human astrocytes. **a** Representative fluorescence micrographs documenting mRFP-EGFP-LC3 expression in mock-infected human astrocytes (Mock) and after exposure to selected flaviviruses (TBEV, WNV, and MOF) for 48 h. Selected rectangular areas within the cells, enlarged at the corners (bottom, right), show superimposed images of mRFP (red) and EGFP (green) fluorescence. Arrows indicate autolysosomes (AL, red-only objects). Adjacent, smaller panels display mRFP and EGFP fluorescence as well as co-localization masks (Col.mask) (co-localized objects correspond to autophagosomes [AP]) of the enlarged images. The white outlines in the large panels show the cell shape. **b** The total number of autophagic compartments (**b**_**i**_) and the ratio of AL to AP (**b**_**ii**_) in mock-infected cells and after infection with TBEV, WNV, and MOF at 12, 24, and 48 h post infection (hpi). Infection with TBEV increases the total number of autophagic compartments compared with mock-infected cells at all three time points tested (*P < 0.05, one-way ANOVA followed by Dunn’s test), but does not affect the AL:AP ratio (*P* > 0.05, one-way ANOVA). WNV infection induces an increase in the total number of autophagic compartments at 48 hpi (*P < 0.05, one-way ANOVA followed by Dunn’s test), and does not affect the ratio AL:AP (*P* > 0.05, one-way ANOVA). MOF infection does not affect either the number of autophagic compartments or the AL:AP ratio compared with mock-infected cells at any time point tested (*P* > 0.05, one-way ANOVA). **c** Diameter of AP and AL in mock-infected cells and 48 hpi with selected flaviviruses. Infection with TBEV, WNV, and MOF does not affect the size of autophagic compartment compared with mock-infected cells (*P* > 0.05, one-way ANOVA). AL are larger than AP in all experimental conditions (***P < 0.001, Mann–Whitney *U* test). Full lines in the boxplots represent median values and the dotted lines correspond to average values. The numbers below the boxplots are the number of cells (**a**, **b**) or compartments (**c**) analyzed for each condition. Cells were infected with TBEV and MOF at an MOI 0.1 and with WNV at an MOI 1

Viral infections can interact with many host cell processes and consequently affect cell functioning. Among others, autophagy is often affected by viruses from different viral families [14, 19]. Therefore, we next investigated whether TBEV, WNV, and MOF affect autophagy in these cells. To this end, human astrocytes were first transfected with the plasmid ptfLC3 to express mRFP-EGFP-LC3 and then infected with the flaviviruses (Fig. 5a). The total number of autophagic compartments (Fig. 5b_i_) and the ratio of autolysosomes to autophagosomes (Fig. 5b_ii_) were determined by confocal microscopy in mock-infected and flavivirus-infected cells at 12, 24, and 48 hpi. Notably, all cells, mock-infected and infected with respective viruses, were maintained in the growth medium for the duration of 48 h (see “Materials and methods” for details). The volume of growth medium (2 ml) was sufficient to prevent the starvation stress over the course of the experiment (Fig. S1b).

TBEV infection induced an increase in the total number of autophagic compartments at all time points (12, 24, and 48 hpi) compared with mock-infected cells (Fig. 5b_i_; *P < 0.05, one-way ANOVA followed by Dunn’s test), without affecting the ratio of autolysosomes to autophagosomes (Fig. 5b_ii_; *P* > 0.05, one-way ANOVA). This indicates that in human astrocytes infected by TBEV, autophagy dynamics is increased early post infection. Among the time points of our measurements, the most prominent TBEV-triggered autophagic response was observed at 48 hpi when cells exhibited an ~ 2.5-fold increase in the number of LC3-positive autophagic compartments compared with mock-infected cells (*P < 0.05, one-way ANOVA followed by Dunn’s test). Interestingly, infection with WNV also resulted in a higher total number of autophagic compartments compared with mock-infected cells; however, the increase was significant only at 48 hpi, when ~ twofold increase was observed (Fig. 5b_i_; *P < 0.05, one-way ANOVA followed by Dunn’s test). Similarly, as in the case of TBEV, infection of astrocytes with WNV did not affect the ratio of autolysosomes to autophagosomes (Fig. 5b_ii_; *P* > 0.05, one-way ANOVA). Infection of cells with MOF did not affect either the total number of autophagic compartments or the ratio of autolysosomes to autophagosomes (Fig. 5b; *P* > 0.05, one-way ANOVA), indicating that MOF does not affect autophagy in human astrocytes. In conclusion, infection of human astrocytes with TBEV and WNV, but not MOF, stimulates autophagy without affecting the autophagosome maturation process.

Next, the diameter of mRFP-EGFP-LC3-labeled autophagic compartments (autophagosomes and autolysosomes) was determined in mock-infected cells at 48 h after infection with selected flaviviruses (TBEV, WNV, and MOF) (Fig. 5c). None of these viruses affected the size of the autophagic compartments, because the average diameter of autophagosomes and autolysosomes was similar in mock-infected cells and in flavivirus-infected cells (*P* > 0.05, one-way ANOVA). Moreover, in all conditions, autolysosomes were larger than autophagosomes (***P < 0.001, Mann–Whitney *U* test).

The diameter of both autophagosomes and autolysosomes in controls measured in confocal micrographs was ~ 10% larger compared with compartments in controls imaged by SIM (the average diameter of autophagosomes and autolysosomes determined by confocal microscopy was 332 nm ± 8 nm (*n* = 133) and 386 nm ± 11 nm (*n* = 112), respectively). The differences observed between the measurements performed with the different fluorescence microscopy techniques likely derive from the limitations of conventional fluorescence microscopy, which enables accurate measurements of structures larger than 200 nm [63]. However, as demonstrated by TEM and SIM, only a small percentage of autophagic compartments have a diameter below this limit.

**Fig. 6:**
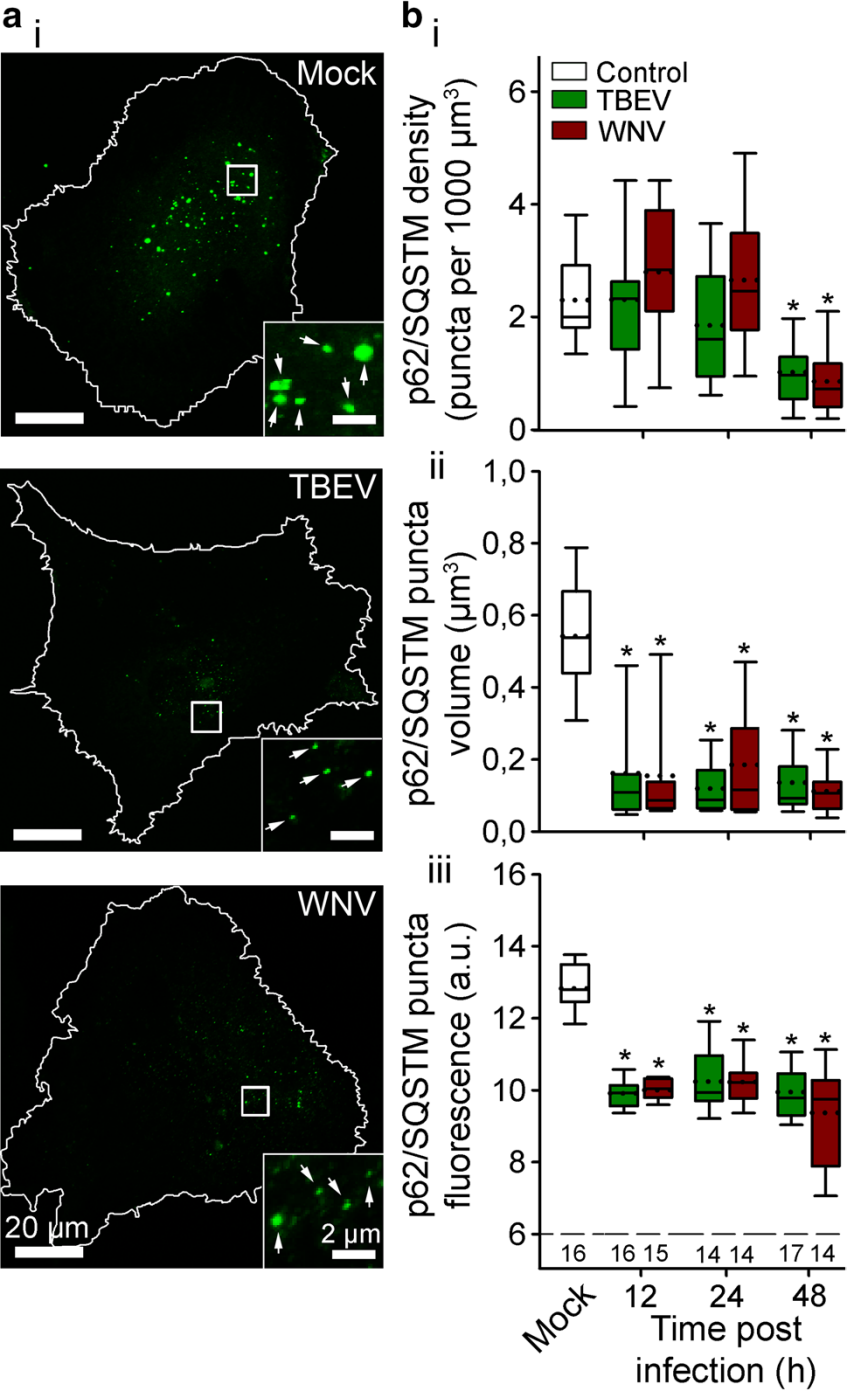
Infection of human astrocytes with tick-borne encephalitis virus (TBEV) and West Nile virus (WNV) decreases the level of p62/SQSTM1, a selective autophagy receptor. **a** Representative fluorescence micrographs showing immuno-labeled p62/SQSTM1 in mock-infected human astrocytes (Mock) and after exposure to TBEV and WNV for 48 h. Selected rectangular areas within the cells, enlarged at the bottom right corners, show fluorescent puncta (p62/SQSTM1), indicated by arrows. Following the infection with TBEV and WNV the density of fluorescent puncta, their size and the intensity of fluorescence is decreased at 48 h post infection (hpi). The white outlines in the large panels show the cell shape. **b** The density (**b**_**i**_), the volume (**b**_**ii**_) and the fluorescence intensity (**b**_**iii**_) of immuno-labeled p62/SQSTM1 in mock-infected astrocytes (Mock) and after infection with TBEV and WNV at 12, 24, and 48 hpi. Infection with TBEV and WNV decreases the density of fluorescent puncta at 48 hpi, whereas the volume and the intensity of fluorescent puncta is decreased at all three time points tested, compared with mock-infected cells (*P < 0.05, one-way ANOVA followed by Dunn’s test). Full lines in the boxplots represent median values and the dotted lines correspond to average values. The numbers below the boxplots in panel **b**_**iii**_ are the number of cells analyzed for each condition. Cells were infected with TBEV at an MOI 0.1 and with WNV at an MOI 1

To further assess the effect of infection of human astrocytes with TBEV and WNV on autophagy, we immuno-labeled p62/SQSTM1 at 12, 24, and, 48 hpi. p62/SQSTM1 was the first selective autophagy receptor discovered in mammals that recruits ubiquitinated proteins and organelles to the autophagosomes [64, 65]. p62/SQSTM1, which is degraded in the process of autophagy, is often used as a marker to monitor autophagy, as its levels are inversely proportional to the autophagic activity [65]. Our results, summarized in Fig. 5, show that infection of human astrocytes with TBEV or WNV stimulates autophagy. In line with these results, the fluorescent signal of p62/SQSTM1, showing punctiform appearance, was visibly less bright in astrocytes infected with TBEV or WNV, compared to mock-infected cells (Fig. 6a). This observation was confirmed also by the analysis, where we measured the density, as well as the volume and the fluorescence intensity of respective p62/SQSTM1 fluorescent puncta. Elevated p62/SQSTM1 degradation, indicated by the decrease of the fluorescence intensity and the volume of fluorescent puncta, was observed already at 12 hpi with TBEV and WNV, respectively (Fig. 6b; *P < 0.05, one-way ANOVA followed by Dunn’s test). Moreover, at 48 hpi also, the density of p62/SQSTM1 puncta was significantly decreased (Fig. 6b; *P < 0.05, one-way ANOVA followed by Dunn’s test), corroborating the results obtained with mRFP-EGFP-LC3 autophagy reporter.

### Rapamycin and wortmannin do not affect the replication of TBEV and WNV, while bafilomycin A1 inhibits it

**Fig. 7:**
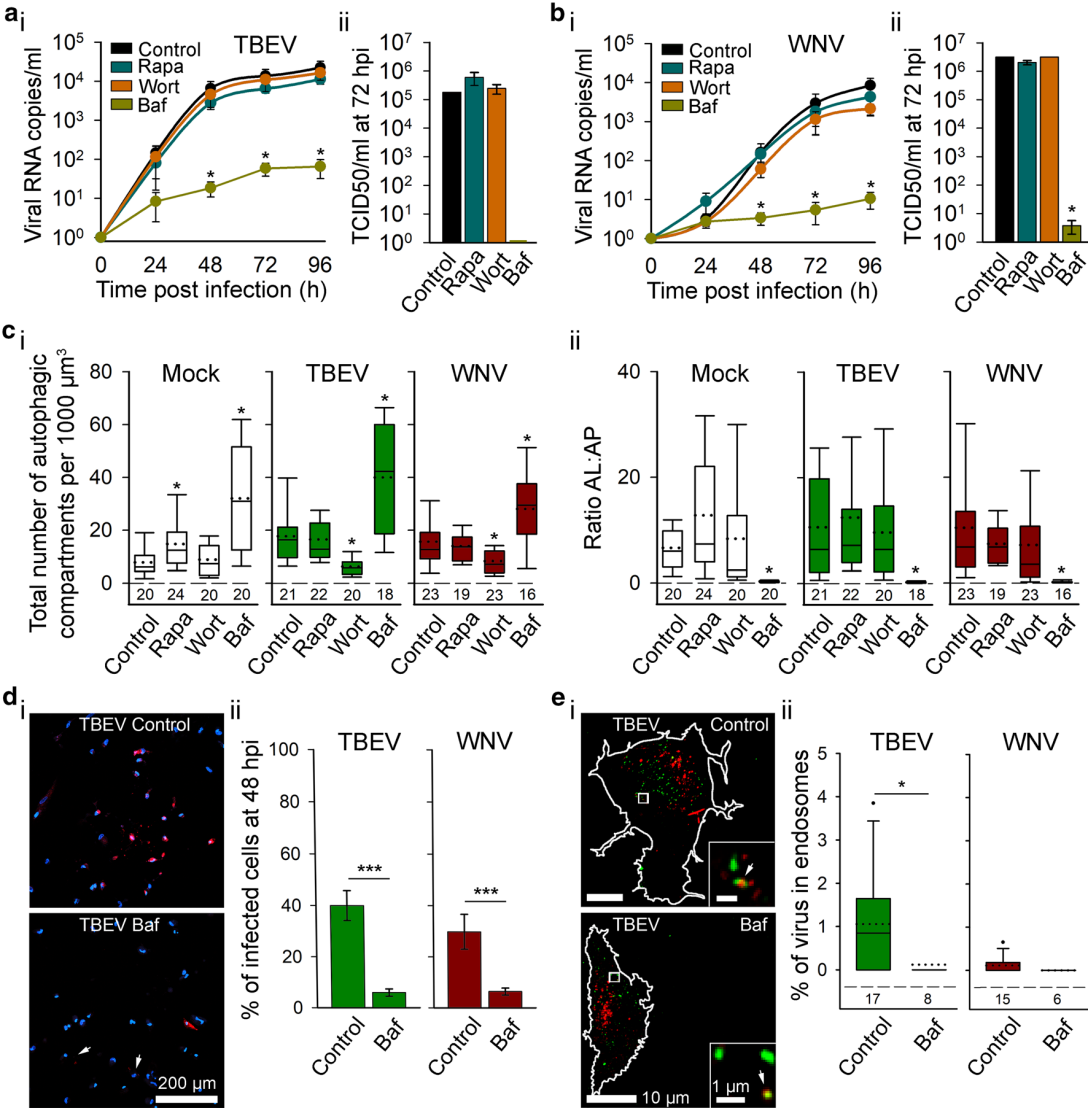
Replication rate of tick-borne encephalitis virus (TBEV) and West Nile virus (WNV) is independent of autophagy, but bafilomycin A1 decreases replication and infectivity of respective viruses in human astrocytes. **a**, **b** Human astrocytes, exposed to pharmacological autophagy modulators (rapamycin (Rapa), wortmannin (Wort) and bafilomycin A1 (Baf)), were inoculated with TBEV (**a**) or WNV (**b**); their replication rates (viral RNA copies/ml) (**a**_**i**_, **b**_**i**_) and the median tissue culture infectious dose (TCID50) (**a**_**ii**_, **b**_**ii**_) is shown at different time points post infection (from 0 to 96 hpi). Treatment of astrocytes with Rapa and Wort does not affect RNA levels of either TBEV or WNV in the cell supernatant at any time point (*P* > 0.05, one-way ANOVA). On the other hand, treatment of astrocytes with Baf reduces TBEV and WNV RNA in the cell culture supernatant from 48 to 96 hpi (*P < 0.05, one-way ANOVA followed by Dunn’s test). Similarly, treatment with Rapa or Wort does not affect the concentration of infectious TBEV (**a**_**ii**_) and WNV (**b**_**ii**_), released from astrocytes at 72 hpi (*P* > 0.05, one-way ANOVA), while the concentration of infectious WNV is significantly decreased following treatment with Baf (*P < 0.05, one-way ANOVA followed by Dunnett’s test). Of note, stimulation with Baf reduces the concentration of infectious TBEV below the limit of detection. Data are presented as an average ± standard error. **c** Autophagy rate in mock-infected (Mock) and flavivirus-infected (TBEV and WNV) human astrocytes in control conditions (Control) and after exposure to Rapa, Wort and Baf at 48 hpi; the total number of autophagic compartments (**c**_**i**_) and the ratio of autolysosomes to autophagosomes (AL:AP; **c**_**ii**_) is shown. Rapa increases the total number of autophagic compartments in mock-infected cells (*P < 0.05, one-way ANOVA followed by Dunn’s test), but not in TBEV- and WNV-infected cells. In contrast, the treatment with Wort results in a lower number of autophagic compartments following infection with TBEV and WNV (*P < 0.05, one-way ANOVA followed by Dunn’s test), but not in mock-infected cells. Baf increases the total number of autophagic compartments in mock-infected, TBEV- and WNV-infected cells (*P < 0.05, one-way ANOVA followed by Dunn’s test). The ratio AL:AP is affected only in Baf-treated cells (mock-infected, TBEV-infected and WNV-infected) (*P < 0.05, one-way ANOVA followed by Dunn’s test). **d**_**i**_ Representative fluorescence micrographs of TBEV-infected cells that were either treated with Baf or not and immuno-labeled with antibodies against flavivirus group antigen (red) at 48 hpi. Cell nuclei are stained with 4′,6-diamidino-2-phenylindole (DAPI; blue). Arrows indicate weakly infected cells. **d**_**ii**_ The percentages of TBEV- and WNV-infected cells at 48 hpi, determined as the ratio between the number of flavivirus-positive cells and the number of DAPI-stained nuclei, were lower in cells treated with Baf (***P < 0.001, Mann–Whitney *U* test). Data are presented as an average ± standard error. The number of cells analyzed for Control and Baf: TBEV (885 and 368), WNV (777 and 249) and of fields of view analyzed for Control and Baf: TBEV (26 and 20), WNV (22 and 20). **e**_**i**_ Representative fluorescence micrographs of TBEV-infected cells that were either treated with Baf or not and immuno-labeled with antibodies against flavivirus group antigen (red) and endosome marker EEA1 (green) at 48 hpi. Selected rectangular areas within the cells, enlarged at the bottom right, show superimposed images of red (TBEV) and green (EEA1) fluorescence. Arrows point to co-localized puncta. Cell outlines are delineated in white. **e**_**ii**_ Treatment of human astrocytes with Baf reduces the percentage of TBEV, but not also of WNV in early endosomes (*P < 0.05, Mann–Whitney *U* test). The numbers below the boxplots are the number of cells analyzed for each condition. Full lines in the boxplots represent median values and the dotted lines correspond to average values. Cells were infected with TBEV at an MOI 0.1 and with WNV at an MOI 1. All statistical comparisons were made against controls

To examine a possible pro-viral role of flavivirus-induced autophagy in human astrocytes, cells were treated with pharmacological autophagy modulators (1 μM Rapa, 100 nM Wort, and 100 nM Baf) and the consequent effect on the dynamics of viral replication was assessed by measuring the concentration of viral RNA in the supernatant of TBEV- or WNV-infected cells (Fig. 7a, b). Furthermore, the infectivity of released virus particles at 72 hpi (time point when viral RNA copies in the cell culture supernatants reached the plateau), was determined by TCID50 assay in the presence or absence of selected autophagy modulators (Fig. 7a, b).

Treatment of astrocytes with modulators of early stages of autophagy, Rapa and Wort, did not affect the concentration of TBEV and WNV RNA in the cell culture supernatant, compared with non-treated controls at any of the tested time points (Fig. 7a_i_, b_i_; *P* > 0.05, one-way ANOVA). In contrast, treatment of astrocytes with Baf resulted in decreased levels of detected viral RNA at 48, 72, and 96 hpi compared with non-treated cells for both flaviviruses (*P < 0.05, one-way ANOVA followed by Dunn’s test). In agreement with these observations, Rapa and Wort did not affect the production of infectious virus particles of TBEV and WNV at 72 hpi compared with non-treated controls (Fig. 7a_ii_, b_ii_; *P* > 0.05, one-way ANOVA). The quantity of infectious virus in Baf-treated astrocytes was below the detection limit in the case of TBEV and significantly reduced in the case of WNV (*P < 0.05, one-way ANOVA followed by Dunnett's test).

To test the efficiency of selected autophagy modulators in virus-infected cells, we transfected astrocytes with ptfLC3, treated them with Rapa, Wort, or Baf, and infected them with TBEV and WNV, respectively, for 48 h (Figs. 7c, S2). In mock-infected cells treatment with Rapa-stimulated autophagy by increasing the total number of autophagic compartments (*P < 0.05, one-way ANOVA followed by Dunn’s test), while Wort did not affect the total number of autophagic compartments and the ratio of autolysosomes to autophagosomes, compared with non-treated controls (*P* > 0.05, one-way ANOVA). As expected, in the absence of autophagy modulators, the total number of autophagic compartments was higher in TBEV- and WNV-infected astrocytes versus mock-infected astrocytes (*P < 0.05, one-way ANOVA followed by Dunn’s test). While treatment of TBEV- and WNV-infected astrocytes with Rapa did not augment autophagy further (*P* > 0.05, one-way ANOVA), treatment with Wort significantly decreased the total number of autophagic compartments (*P < 0.05, one-way ANOVA followed by Dunn’s test) to levels comparable with mock-infected controls, indicating that Wort prevents virus-induced autophagy at 48 hpi. Treatment of astrocytes with Baf significantly increased the total number of autophagic compartments and decreased the ratio of autolysosomes to autophagosomes in all tested conditions (Mock, TBEV, and WNV) (*P < 0.05, one-way ANOVA followed by Dunn’s test).

To further assess the effect of Baf on TBEV- and WNV-infected astrocytes, we determined the infection rates in non-treated and Baf-treated cells at 48 hpi (Fig. 7d). Treatment with Baf significantly decreased the percentages of infected human astrocytes for both viruses (Fig. 7d_ii_; ***P < 0.001, Mann–Whitney *U* test), in agreement with decreased RNA replication and infectious virus production in Baf-treated human astrocytes. Baf is a specific inhibitor of V-ATPase proton pumps [58, 59], and thus prevents acidification of different V-ATPase containing intracellular vesicles, including endosomes [66, 67]. As flaviviruses predominantly enter the cell through receptor-mediated endocytosis [27], they are initially enclosed in endosomes. The residence time of viruses inside an endosome depends on endosome acidification, as acidic intraluminal pH is needed for conformational rearrangement of viral glycoproteins and fusion of viral and endosomal membranes [27, 68]. To test whether TBEV and WNV remain entrapped in early endosomes of human astrocytes, we next evaluated co-localization between immuno-labeled viral particles and EEA1-positive endosomes in non-treated and Baf-treated cells at 48 hpi (Fig. 7e). Interestingly, our results demonstrate that following the treatment with Baf, the percentage of virus particles in endosomes decreases in the case of TBEV (Fig. 7e_ii_; *P < 0.05, Mann–Whitney *U* test) and remains unchanged in the case of WNV (Fig. 7e_ii_; *P* > 0.05, Mann–Whitney *U* test).

## Discussion

### Starvation and pharmacological modulators alter autophagy dynamics and autophagic compartment sizes in human astrocytes

Since the discovery of macro-autophagy in the late 1950s (for a detailed historical overview, see Ohsumi [69]), a major effort has been made to develop methodological approaches to study different intermediates of the autophagic pathway. In particular, the multistep nature of autophagy increases the need to evaluate autophagy dynamics, including the rate of conversion between different autophagic compartments [49, 50, 70, 71]. In this study, we show that plasmid ptfLC3, encoding tandem fluorescent-tagged LC3 (mRFP-EGFP-LC3) [46], in combination with different fluorescence microscopy techniques enables robust measurements of autophagic activity and the size of autophagic compartments in human astrocytes.

We validated the efficacy of the mRFP-EGFP-LC3 probe in human astrocytes by exposing them to EBSS, a medium mimicking nutrient and growth factor deprivation, and to selected pharmacological autophagy modulators. Assessment of autophagic activity was based on measurements of the number of LC3-positive autophagic compartments and on the ratio of autolysosomes to autophagosomes in individual mRFP-EGFP-LC3-expressing cells. Starvation conditions increased the rate of autophagy in human astrocytes, as previously observed in cultured murine and sheep astrocytes [72–74], as well as in many other cell types [75–78]. Modulation of autophagy with established pharmacological modulators of autophagy, i.e., Rapa, Wort, and Baf, further validated the use of mRFP-EGFP-LC3 in human astrocytes. Specifically, Rapa induced an increase in the number of LC3-positive autophagic compartments, Wort prevented Rapa-induced autophagy, and treatment with Baf altered the ratio of autolysosomes to autophagosomes in favor of autophagosomes. These observations are in agreement with accepted mechanisms of action of the respective pharmacological modulators [50, 79].

Furthermore, we report that human astrocytes exhibit a relatively high rate of autophagy at rest, because numerous LC3-positive compartments were observed in cells maintained in nutrient-rich conditions. This observation is in line with reports on some other cell types, such as liver cells, podocytes, lens epithelial cells, and post-mitotic cells (such as neurons), where high constitutive autophagy is likely needed to cope with high energy turnover and/or to provide efficient quality control by constitutively removing long-lived and damaged cellular components [76, 80, 81]. Moreover, a group of researchers previously demonstrated a high rate of autophagy in astrocytes, based on western blot measurements of the levels of LC3-II and Beclin 1 [82]. However, the number of LC3-positive compartments further increased under starvation conditions and after treatment with Rapa in our experimental conditions, indicating that astrocytes are capable of increasing autophagy when necessary.

Using mRFP-EGFP-LC3, we cannot distinguish between all autophagic intermediates, i.e., phagophores, autophagosomes, amphisomes, and autolysosomes [50]. The lipidated form of LC3 (termed LC3-II) integrates in the inner and outer autophagic membranes during phagophore elongation and remains attached until degradation in autolysosomes (although LC3 is released to some extent from the outer autophagosome membrane during the maturation process) [3, 48, 83], indicating that LC3 marks a number of autophagy intermediates. In our analysis, we assumed that all mRFP^+^EGFP^+^ objects correspond to autophagosomes, and all mRFP^+^EGFP^−^ objects denote autolysosomes. However, a proportion of the mRFP^+^EGFP^+^ objects most likely correspond to phagophores, the first intermediates of the autophagy pathway, all of which at some point become autophagosomes. Similarly, some of the mRFP^+^EGFP^−^ structures may correspond to amphisomes, because fusion of autophagosomes with late endosomes also results in a decrease in pH [84], which creates conditions where EGFP fluorescence is possibly quenched [46].

Next, we report that in human astrocytes, autophagic compartments vary considerably in size; structures with diameter of 157–1545 nm were observed in normal growth conditions (nutrient-rich medium), as monitored by super-resolution microscopy. The mean diameter of autolysosomes was ~ 15% larger than that of autophagosomes. Previous reports on the diameter of autophagic compartments, obtained mostly by electron microscopy (EM), state values between 200 and 900 nm in yeast cells [85, 86] and values between 500 and 1500 nm in mammalian cells [5, 87, 88]. In our measurements, 93% of autophagic compartments (autophagosomes and autolysosomes) were smaller than 500 nm. Similar observations were reported for neurons, where more than 90% of autophagic compartments were smaller than 750 nm, as measured by a fluorescent reporter system [89]. A possible explanation for the relatively larger EM-based measurements is that bigger structures are more easily recognized on ultrathin sample slices and thus more likely analyzed [86]. In agreement, in our study, the diameter of autophagic compartments determined by TEM was larger than the values obtained by SIM. Specifically, the average diameter of autophagic compartments determined by TEM in control resting conditions was 449 nm ± 29 nm, whereas SIM measurements yielded an average diameter of 304 nm ± 6 nm and 355 nm ± 6 nm for autophagosomes and autolysosomes, respectively. Fluorescence microscopy techniques (SIM and confocal microscopy) have two advantages over EM when monitoring autophagic compartments. First, they allow optical sectioning of cells, which enables assessment of cellular structures within the whole cell as opposed to EM, where relatively small cellular sections are observed [90]. Second, fluorescent reporters, such as mRFP-EGFP-LC3, facilitate identification of autolysosomes, whereas in EM slices, autolysosomes are difficult to distinguish from other membranous organelles (in contrast to autophagosomes, which can be easily recognized due to the double-membrane morphology) [49, 90]. Consequently, clear discrimination between autolysosomes and autophagosomes observed by fluorescence microscopies enables accurate determination of autophagy dynamics, which is not possible by EM. One major advantage of EM over any fluorescence microscopy technique, even super-resolution techniques, is the superior resolution. Considering that the smallest autophagic compartment had a diameter of 155 nm, as determined by TEM, this advantage is not pertinent in this particular case, because structures of this size are clearly discernible with SIM. Moreover, reported sizes of autophagic compartments are often determined in stimulated conditions, which ensure a high rate of autophagy, but can also induce formation of larger autophagic compartments compared with non-stimulated conditions [91]. For example, a starvation-induced increase in the size of autophagosomes was observed in multiple mice tissues [76, 78]. Our results support these observations, because autolysosomes were larger in starved and Rapa-treated human astrocytes compared with non-stimulated conditions. Interestingly, treatment of astrocytes with Rapa also affected the diameter of autophagosomes, whereas starvation did not, which could indicate these two stimulators exploit different signaling pathways to regulate the autophagic response.

### TBEV and WNV efficiently infect and replicate in human astrocytes in a time-dependent manner, whereas MOF displays low infectivity

Measurements of the infection rates, RNA amplification dynamics and production of infectious viral particles confirmed that TBEV and WNV infect and replicate in human astrocytes, which is in line with previously demonstrated astrocyte susceptibility to infection with various neurotropic flaviviruses, including TBEV [24, 33, 41], WNV [38, 92, 93], JEV [94, 95], and ZIKV [34, 36, 43]. At 48 hpi, when the RNA replication rate for both viruses (TBEV and WNV) and the infectious virus production from TBEV- or WNV-infected human astrocytes reached a plateau, the infection rate of TBEV was approximately 20% higher than that of WNV. In addition, we report for the first time that MOF isolated from mosquito species *Aedes albopictus* [44], which belongs to a group of viruses without a known vertebrate host and are considered non-pathogenic, as the name of these viruses implies [39], can actually infect human astrocytes. At present, the mechanism of MOF entry into cells is not known; i.e., it is not clear if MOF enters cells via a yet unidentified attachment factor located on the surface of astrocytes, followed by endocytosis, or via pinocytosis. Alternatively, detected MOF signals could correspond to MOF particles non-specifically bound to the plasma membrane. However, the time-dependent increase in the percentage of infected cells, albeit remaining relatively low throughout the observation period (reaching a maximum of 4% at 48 hpi), renders this explanation unlikely. A related question is what happens with MOF after its presumed entry into astrocytes. MOF RNA levels in the supernatant remained very low throughout the experiment. This indicates that MOF either did not replicate in astrocytes and that the RNA detected represents unwashed virus, or that its replication was at best very modest. Similarly, we could not detect any infectious virus production from MOF-infected human astrocytes. Our results are in line with previous findings that various MOFs replicate in mosquito cells but not in mammalian cells [96–98]. Nevertheless, our results show that astrocytes are somewhat susceptible to MOF infection; however, we do not assume that MOF presents a threat to human health. Viruses must invade the CNS to infect astrocytes and other types of neural cells. Flaviviruses typically replicate locally and in regional lymph nodes, resulting in viremia, which can be maintained only if there is continuing introduction of virus into the bloodstream from infected tissues to counter the continual removal of virus by macrophages and other cells and the natural decay of virus infectivity over time [99]. Because MOF is not capable of efficient replication in vertebrate cells, the occurrence of high viremia and/or breaching of the BBB is a highly unlikely event. However, the importance of MOFs may be their ability to drive the evolution of medically important arthropod-borne flaviviruses [96]. Recently, we have been witnessing increased awareness of a high prevalence and biodiversity of MOFs in nature; and these findings should drive future research on MOFs to increase our current very limited understanding of their significance and implications for their evolution and transmission [100]. Clearly, further research is warranted to explore the possibility of MOF replication in vertebrate cells.

### TBEV and WNV trigger autophagic response in human astrocytes, but the replication of these viruses is independent of autophagy

In human astrocytes, infection with TBEV and WNV induced an increase in the number of autophagic compartments without affecting the ratio of autolysosomes to autophagosomes, suggesting that autophagy is augmented while the autophagosome maturation process remains intact in flavivirus-infected astrocytes. The rate of autophagy following infection was comparable to Rapa-stimulated conditions and Wort efficiently prevented virus-triggered autophagy increase, which is in line with the view that TBEV and WNV activate autophagy through canonical mechanism of autophagy (PtdIns3K-dependent mechanism). In agreement, infection with TBEV and WNV decreased levels of p62/SQSTM1, which acts as a substrate during autophagic degradation. These observations are consistent with findings on other mammalian cell types, since the majority of reports associate flavivirus infections with undisturbed autolysosome formation and functional autophagic degradation [19, 101–104]. Induction of autophagy by TBEV was more prominent than by WNV, as the observed autophagic response, measured by the ptfLC3 autophagy reporter, was faster (observed already at 12 hpi) and the number of detected LC3-positive autophagic compartments at 48 hpi was ~ 20% higher. These effects could be related to higher infectivity and faster production of infectious TBEV in human astrocytes compared with WNV and/or could be attributed to distinct regulation of autophagy by respective viruses. Infection with TBEV has been shown to deplete AKT levels, which induces autophagy by inhibiting the AKT-mTOR pathway [105]. On the other hand, the observed delay in augmentation of autophagy upon WNV infection could be due to indirect mechanisms of autophagy activation. Specifically, autophagy can be initiated by other cellular stress pathways, such as endoplasmic reticulum stress and unfolded protein response [106, 107]. Modulation of the pathways of the unfolded protein response is a characteristic of many RNA viruses [108–110] and has been previously confirmed for WNV in human neural cells [111]. In our experiments, the rate of p62/SQSTM1 degradation, following the infection with TBEV and WNV, was similar. However, the intracellular level of p62/SQSTM1 depends not only on autophagic degradation, but also on transcriptional regulation. The latter is affected by several factors; e.g., p62/SQSTM1 transcription is induced by NF-E2-related factor 2 (NRF2) during oxidative stress [112]. In contrast to WNV infection, which does not lead to characteristic oxidative stress, TBEV has been shown to activate NRF2 pathway [113]. Consequently, the upregulation of p62/SQSTM1 expression during the TBEV infection may partly mask the difference in p62/SQSTM1 degradation, compared to the p62/SQSTM1 degradation during WNV infection. In line with the low extent of viral replication and release, MOF infection does not affect autophagy in human astrocytes.

Although autophagy is considered a primordial mechanism of antiviral defense, it appears to have the opposite function in the case of the pathogenesis of many RNA viruses, including flaviviruses [18, 101, 102, 114, 115]. For example, virus-triggered autophagy facilitates dengue virus replication and growth in many cell lines [116, 117] and supports JEV replication in early stages of infection [118]. To better understand possible involvement of astrocytic autophagy in life cycle of pathogenic flaviviruses, we examined the TBEV and WNV replication rate and infectious virus production in the presence of autophagy modulators. Rapa (a classic activator of autophagy) and Wort (an inhibitor of early stages of autophagy) did not affect the viral replication dynamics or efficiency of infectious virus release for either TBEV or WNV, indicating that autophagy is not required for flavivirus replication in human astrocytes. Similarly, several reports have suggested that autophagy modulation does not influence WNV replication [104, 119]. In contrast, TBEV replication and growth can depend on autophagy modulation as previously demonstrated in human neuroblastoma cells [120]. In short, interactions between flavivirus life cycle and autophagy may be cell type-specific.

Interestingly, treatment of astrocytes with Baf, which is an inhibitor of late stages of autophagy, resulted in significantly altered parameters of flavivirus infections; it reduced the amounts of TBEV and WNV RNA in the cell culture supernatant, completely abolished and strongly inhibited the astrocyte-mediated production of infectious TBEV and WNV, respectively, and decreased the rate of infection of cultured human astrocytes. This could imply that late autophagy events, such as autophagosome maturation and autolysosome acidification, regulate infectivity of flaviviruses. Treatment with Baf can also result in autophagy-independent effects, since it prevents acidification of endosomes, an important organelle in the life cycle of flaviviruses [27]. Therefore, co-localization between viruses and endosomes was evaluated in the presence of Baf. However, Baf treatment did not lead to entrapment of virus in endosomes, but had the opposite effect, as it decreased the amount of virus in endosomes in the case of TBEV. Of note, the observed amount of viral particles in endosomes in non-treated astrocytes was relatively low at 48 hpi, compared with previous studies [33]. Virus entry, trafficking and replication are highly dynamic processes; e.g., the infection of cultured rat astrocytes with TBEV peaks at ~ 18 hpi and the presence of TBEV in endosomes increases from 4 to 18 hpi [33]. WNV has even shorter latent period, as the accumulation of a large number of virus-containing late endosomes and lysosomes was observed within ~ 1 hpi in Vero cells [121]. Nevertheless, if the predominant mechanism, by which Baf reduces the astrocyte-mediated production of infectious TBEV and WNV, was associated with the entrapment of viruses in endosomes, accumulation of viral particles in endosomes should be observed also at 48 hpi. In short, our results suggest that in human astrocytes Baf-mediated inhibition of flavivirus replication and spread is not a consequence of diminished release of viral nucleocapsid from endosomes into the cytoplasm. Possibly, Baf may inhibit early stages of viral infections, such as viral entry, which was recently demonstrated for ZIKV infection of human cell lines [122, 123]. Alternatively, Baf could inhibit viral replication through autophagy-related mechanisms. There are some reports indicating late autophagy events can regulate viral infections. Specifically, l-asparagine-induced inhibition of fusion between autophagosomes/amphisomes and lysosomes decreases production of dengue virus 3 in human hepatoma cell line [124], and complete autolysosomal maturation is necessary for hepatitis C virus genome replication and expression of viral proteins [110].

In summary, our results indicate that TBEV and WNV replicate independently of autophagy in human astrocytes, which mitigates a pro-viral role of autophagy in human astrocytes infected with these viruses. Nevertheless, virus-induced enhancement of autophagy may benefit the virus by interfering with other viral or host cell processes. For example, in the case of dengue virus and ZIKV, involvement of autophagy in viral particle maturation and packaging was recently demonstrated [114, 125]. Induction of autophagy can act favorably for infection also by postponing cell death of infected cells [102, 115]. Indeed, expression of flavivirus protein NS4A was shown to induce autophagy and prevent cell death of epithelial cells, which results in virtually persistent flavivirus infection in certain cell types [126]. In addition, viruses can also use autophagy for shielding from immune sensors and/or regulating innate immune response, as shown for hepatitis C virus [110].

In conclusion, we show for the first time that in addition to TBEV and WNV, also MOF infects human astrocytes, although to a lesser extent. In contrast to TBEV and WNV, MOF is most likely unable to replicate efficiently in this cell type. In human astrocytes, the infectivity of pathogenic flaviviruses (TBEV and WNV) is mirrored by increased rate of autophagy. Conversely, flavivirus replication is independent of autophagy in this cell type. In addition, Baf displays strong antiviral effect in replication and infectivity of pathogenic flaviviruses in astrocytes.

## Supplementary Information

Below is the link to the electronic supplementary material.

Supplementary file1 (DOCX 1197 kb)

## Data Availability

All data that support the findings of this study are published in the article and are available from the corresponding author upon reasonable request.

## Abbreviations

Baf: Bafilomycin A1
BBB: Blood–brain barrier
CNS: Central nervous system
DAPI: 4′,6-Diamidino-2-phenylindole
EBSS: Earle’s balanced salt solution
EGFP: Enhanced green fluorescent protein
EM: Electron microscopy
FBS: Fetal bovine serum
FKBP12: FK506-binding protein
hpi: Hours post infection
JEV: Japanese encephalitis virus
LC3: Microtubule associated protein 1 light chain 3
MOF: Mosquito-only flavivirus
MOI: Multiplicity of infection
mRFP: Monomeric red fluorescent protein
mTORC1: Mammalian target of rapamycin complex 1
PBS: Phosphate-buffered saline
PtdIns3K: Phosphoinositide 3-kinase
Rapa: Rapamycin
RT-PCR: Reverse transcription polymerase chain reaction
SIM: Structured illumination microscopy
TCID50: Median tissue culture infectious dose
TBEV: Tick-borne encephalitis virus
TEM: Transmission electron microscopy
WNV: West Nile virus
Wort: Wortmannin
ZIKV: Zika virus
