# Supplementary material for: In human astrocytes neurotropic flaviviruses increase autophagy, yet their replication is autophagy-independent
Source: Cell Mol Life Sci. 2022 Oct 25;79(11):566. doi: 10.1007/s00018-022-04578-7 (PMC9596533; doi:10.1007/s00018-022-04578-7)
Supplement: Supplementary file 1 — Supplementary file1 (DOCX 1197 kb) [file 18_2022_4578_MOESM1_ESM.docx]

# Electronic supplementary material

In human astrocytes neurotropic flaviviruses increase autophagy, yet their replication is autophagy-independent

Petra Tavčar Verdev^1^, Maja Potokar^1,2^, Miša Korva^3^, Katarina Resman Rus^3^, Marko Kolenc^3^, Tatjana Avšič Županc^3^, Robert Zorec^1,2^, Jernej Jorgačevski^1,2^

^1^Laboratory of Neuroendocrinology – Molecular Cell Physiology, Institute of Pathophysiology, Faculty of Medicine, University of Ljubljana, Ljubljana, Slovenia

^2^Celica Biomedical, Ljubljana, Slovenia

^3^Institute of Microbiology and Immunology, Faculty of Medicine, University of Ljubljana, Ljubljana, Slovenia

Corresponding author: Jernej Jorgačevski [jernej.jorgacevski@mf.uni-lj.si](mailto:jernej.jorgacevski@mf.uni-lj.si) (ORCID 0000-0003-3550-2011)

Journal name: Cell and Molecular Life Sciences

# Supplementary materials and methods

## Cell culture of rat astrocytes

Primary astrocyte cell cultures were prepared from the cerebral cortex of 2‑3 days old female Wistar rats. The care of experimental animals and euthanasia of animals were performed in accordance with the following ethical codes and directives: International Guiding Principles for Biomedical Research Involving Animals developed by the Council for International Organizations of Medical Sciences and the Directive on Conditions for Issue of License for Animal Experiments for Scientific Research Purposes (Official Gazette of the Republic of Slovenia 40/85, 22/87, 43/07). The protocol for the euthanasia of animals used in our study was approved by the Veterinary Administration of the Ministry for Agriculture and the Environment of the Republic of Slovenia (permit no. U34401-26/2020/4), issued on 2 December 2020. After brain isolation, cerebral cortex tissue was extracted and brain meninges were removed. Remaining tissue was suspended in isolation medium (Leibovitz’s L-15 medium supplemented with 2 mM L-glutamine, 5 U/ml penicillin, 5 µg/ml streptomycin, and 1 mg/ml bovine serum albumin) and centrifuged twice at room temperature (4 min at 1200 rpm/min) in a Centric 332A centrifuge (Tehtnica, Slovenija). The pellet was suspended in fresh isolation medium, filtered (pore diameter of 70 μm; Militenyi Biotec, 130-095-823) and centrifuged one more time. Remaining material was plated on cell culture growing flasks and maintained in astrocyte growth medium (high-glucose Dulbecco’s modified Eagle’s medium supplemented with 10% fetal bovine serum, 1 mM sodium pyruvate, 2 mM L-glutamine, 5 U/ml penicillin, and 5 µg/ml streptomycin) at 37°C, 5% CO_2_ atmosphere, and 95% relative humidity. Sub-confluent cultures were shaken at 225 rotations per minute overnight (repeated 3 times) to ensure astrocyte enrichment and further maintained in growth medium at 37°C, 5% CO_2_ atmosphere, and 95% relative humidity. For experiments, cells were plated onto 22-mm diameter glass coverslips coated with poly-D-lysine.

# Supplementary figures and captions


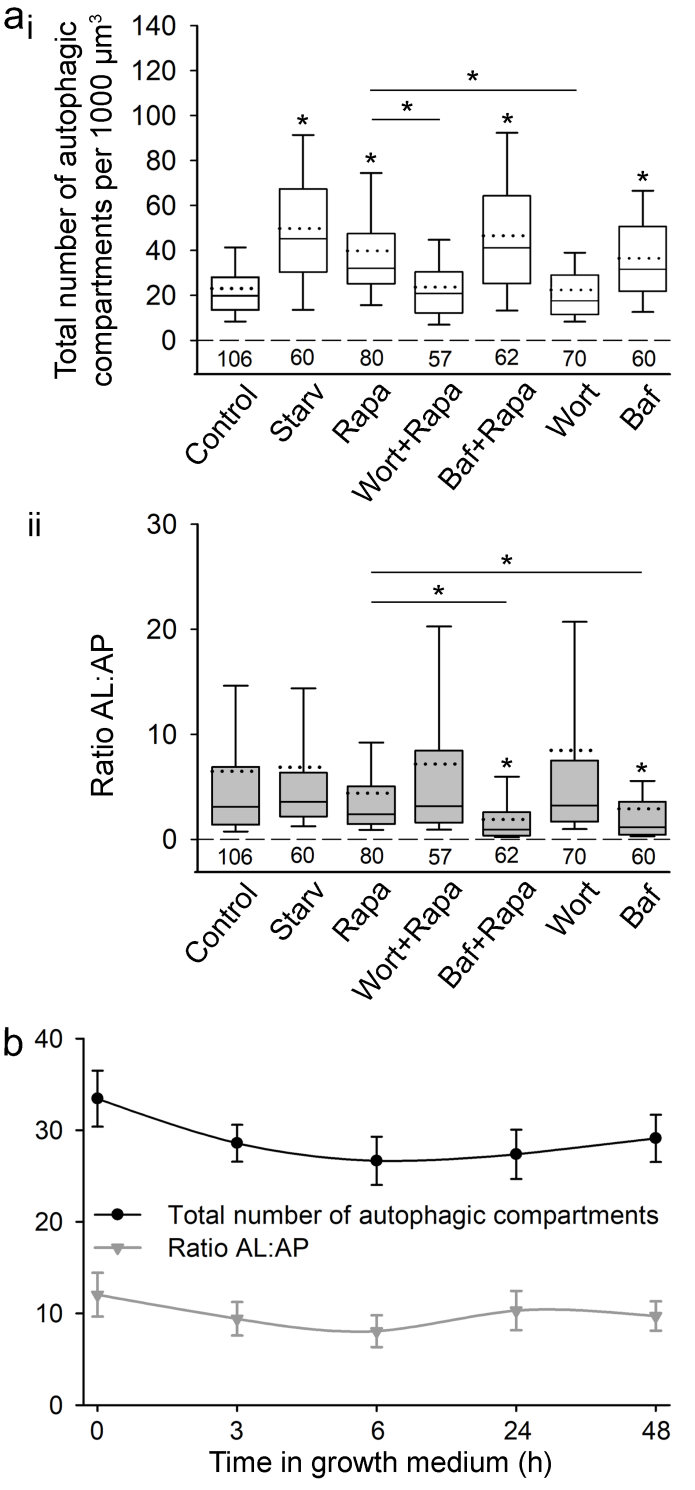


**Fig. S1** Autophagy dynamics in ptfLC3-transfected primary rat astrocytes. **a** The total number of autophagic compartments (**a_i_**) and the ratio of autolysosomes to autophagosomes (AL:AP; **a_ii_**) in ptfLC3-expressing control cells (maintained in a growth medium for 3 h; Control), starved cells (maintained in a medium devoid of nutrients and serum for 3 h; Starv) and cells treated with selected autophagy modulators for 3 h (rapamycin (Rapa), wortmannin (Wort), bafilomycin A1 (Baf)). Starv and Rapa both increase the total number of autophagic compartments (**P*< 0.05, one-way ANOVA followed by Dunn’s test) without affecting the ratio AL:AP (*P*> 0.05, one-way ANOVA) versus non-treated controls. Treatment with Baf+Rapa and Baf increases the total number of autophagic compartments and induces a decrease in the ratio AL:AP (**P*< 0.05, one-way ANOVA followed by Dunn’s test), compared with non-treated controls. Treatment with Wort+Rapa and Wort results in a lower number of autophagic compartments versus Rapa (**P*< 0.05, one-way ANOVA followed by Dunn’s test) and treatment with Baf+Rapa and Baf decreases the ratio AL:AP compared with Rapa-treated cells (**P*< 0.05, one-way ANOVA followed by Dunn’s test). Full lines in the boxplots represent median values and dotted lines correspond to average values. The numbers below the boxplots are the number of cells analyzed for each condition **b** 2 ml of growth medium contains sufficient amount of nutrients to avoid augmentation of the autophagy rate in rat astrocytes within 48 h. Following transfection of cultured rat astrocytes (1.5×10^4^ cells per coverslip) with ptfLC3 for 48 h, the growth medium was exchanged with a fresh one. Samples were then chemically fixed at five different time points after the exchange of the growth medium (0 h, 3 h, 6 h, 24 h, and 48 h) and the autophagy rate was determined based on measurements of the total number of autophagic compartments per 1000 µm^3^ (black curve) and the ratio AL:AP (grey curve). The addition of fresh growth medium did not affect autophagy levels at any time point tested (*P* > 0.05, one-way ANOVA versus 0 time point). Data are presented as an average ± standard error. The number of cells analyzed: 0 h (51), 3 h (55), 6 h (39), 24 h (38), 48 h (32)


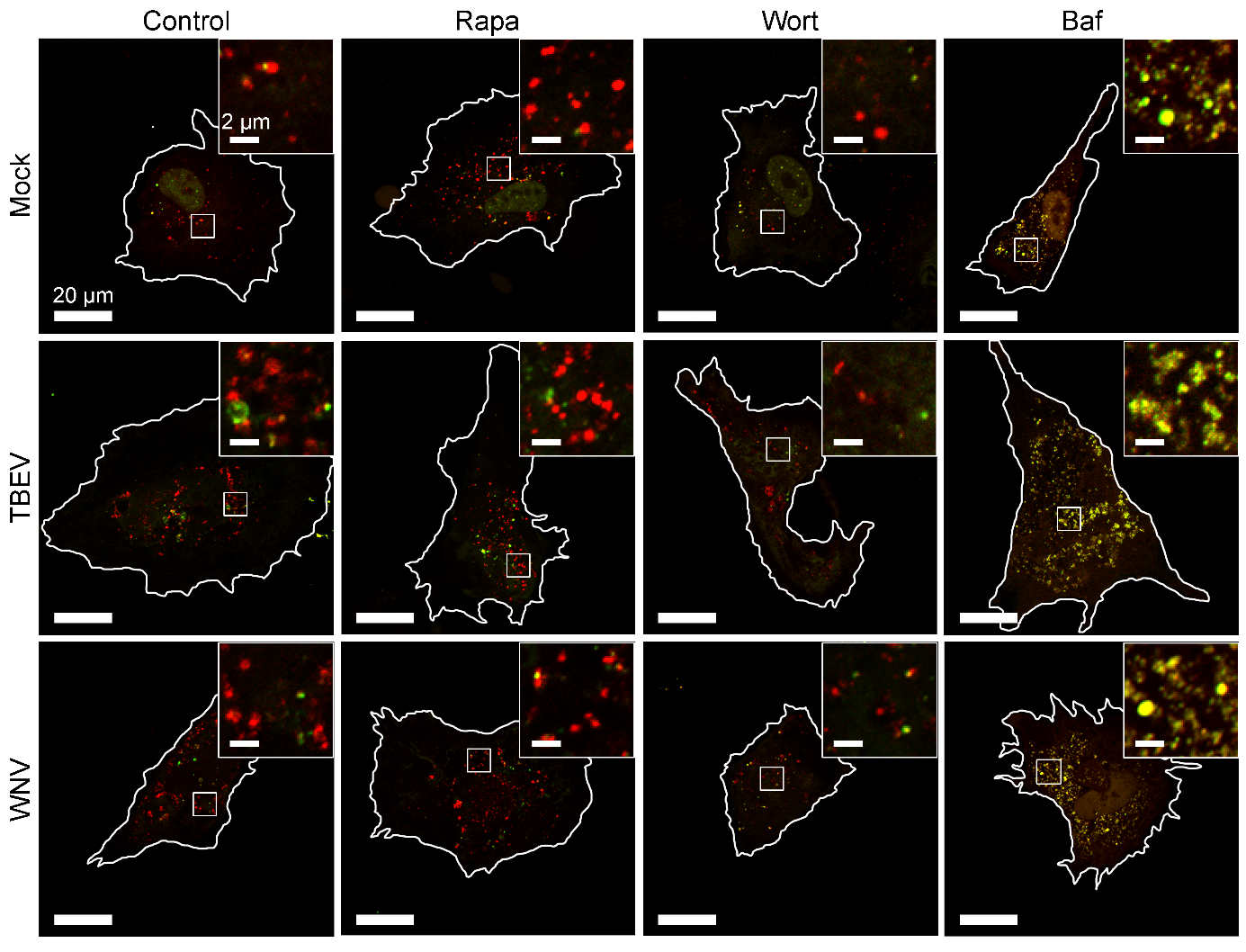


**Fig. S2** Representative fluorescent micrographs showing mRFP-EGFP-LC3 expression in mock-infected (Mock) and flavivirus-infected (TBEV and WNV) human astrocytes, maintained in control conditions (in the absence of autophagy modulators; Control) or exposed to selected autophagy modulators (Rapa, Wort, Baf) for 48 h. Selected rectangular areas within the cells are enlarged at the top right corners showing superimposed images of mRFP (red) and EGFP (green) fluorescence. Autophagosomes are recognized as mRFP^+^EGFP^+^ objects, whereas autolysosomes correspond to mRFP^+^EGFP^-^ objects. The white outlines in the large panels show the cell shape. Cells were infected with TBEV at an MOI 0.1 and WNV at an MOI 1. Cells were exposed to viruses and autophagy modulators for 48 h
